# Mechanistic differences between HIV-1 and SIV nucleocapsid proteins and cross-species HIV-1 genomic RNA recognition

**DOI:** 10.1186/s12977-016-0322-5

**Published:** 2016-12-29

**Authors:** Klara Post, Erik D. Olson, M. Nabuan Naufer, Robert J. Gorelick, Ioulia Rouzina, Mark C. Williams, Karin Musier-Forsyth, Judith G. Levin

**Affiliations:** 1 Section on Viral Gene Regulation, Program in Genomics of Differentiation, Eunice Kennedy Shriver National Institute of Child Health and Human Development, National Institutes of Health, Bethesda, MD 20892-2780 USA; 2 Department of Chemistry and Biochemistry, Center for Retrovirus Research, and Center for RNA Biology, The Ohio State University, Columbus, OH 43210 USA; 3 Department of Physics, Northeastern University, Boston, MA 02115 USA; 4 AIDS and Cancer Virus Program, Leidos Biomedical Research, Inc., Frederick National Laboratory for Cancer Research, Frederick, MD 21702-1201 USA

**Keywords:** HIV-1, SIV, Nucleocapsid proteins, Nucleic acid chaperone activity, Psi RNA packaging signal, Minus-strand transfer, Small angle X-ray scattering, Single molecule DNA stretching, Nucleic acid binding, Reverse transcription

## Abstract

**Background:**

The nucleocapsid (NC) domain of HIV-1 Gag is responsible for specific recognition and packaging of genomic RNA (gRNA) into new viral particles. This occurs through specific interactions between the Gag NC domain and the Psi packaging signal in gRNA. In addition to this critical function, NC proteins are also nucleic acid (NA) chaperone proteins that facilitate NA rearrangements during reverse transcription. Although the interaction with Psi and chaperone activity of HIV-1 NC have been well characterized in vitro, little is known about simian immunodeficiency virus (SIV) NC. Non-human primates are frequently used as a platform to study retroviral infection in vivo; thus, it is important to understand underlying mechanistic differences between HIV-1 and SIV NC.

**Results:**

Here, we characterize SIV NC chaperone activity for the first time. Only modest differences are observed in the ability of SIV NC to facilitate reactions that mimic the minus-strand annealing and transfer steps of reverse transcription relative to HIV-1 NC, with the latter displaying slightly higher strand transfer and annealing rates. Quantitative single molecule DNA stretching studies and dynamic light scattering experiments reveal that these differences are due to significantly increased DNA compaction energy and higher aggregation capability of HIV-1 NC relative to the SIV protein. Using salt-titration binding assays, we find that both proteins are strikingly similar in their ability to specifically interact with HIV-1 Psi RNA. In contrast, they do not demonstrate specific binding to an RNA derived from the putative SIV packaging signal.

**Conclusions:**

Based on these studies, we conclude that (1) HIV-1 NC is a slightly more efficient NA chaperone protein than SIV NC, (2) mechanistic differences between the NA interactions of highly similar retroviral NC proteins are revealed by quantitative single molecule DNA stretching, and (3) SIV NC demonstrates cross-species recognition of the HIV-1 Psi RNA packaging signal.

**Electronic supplementary material:**

The online version of this article (doi:10.1186/s12977-016-0322-5) contains supplementary material, which is available to authorized users.

## Background

AIDS, a devastating disease that emerged in the late twentieth century, is caused by two lentiviruses: HIV-1 [1–3] and HIV-2 [4]. Early on, there was intense interest in the origin of these viruses and the AIDS pandemic, which by 2014 led to infection of 76.2 million people (UNAIDS 2014 estimates, P.D. Ghys, personal communication). Molecular and phylogenetic analyses of fecal samples collected from the forest floor, primarily in southern Cameroon, demonstrated that HIV-1 infection of humans resulted from cross-species transmission of a chimpanzee simian immunodeficiency virus (SIVcpz), a recombinant generated from two distinct monkey SIV lineages [5, 6] (reviewed in [7, 8]). HIV-2 was transmitted to humans by an SIV from sooty mangabeys (SIVsm) [9–11]. Interestingly, SIVmac [12, 13], a strain used in non-human primate model systems (including the present work), was acquired unexpectedly by transmission of SIVsm from sooty mangabeys to rhesus macaques at the California National Primate Research Center, where both groups of animals were housed [14, 15].

**Fig. 1:**
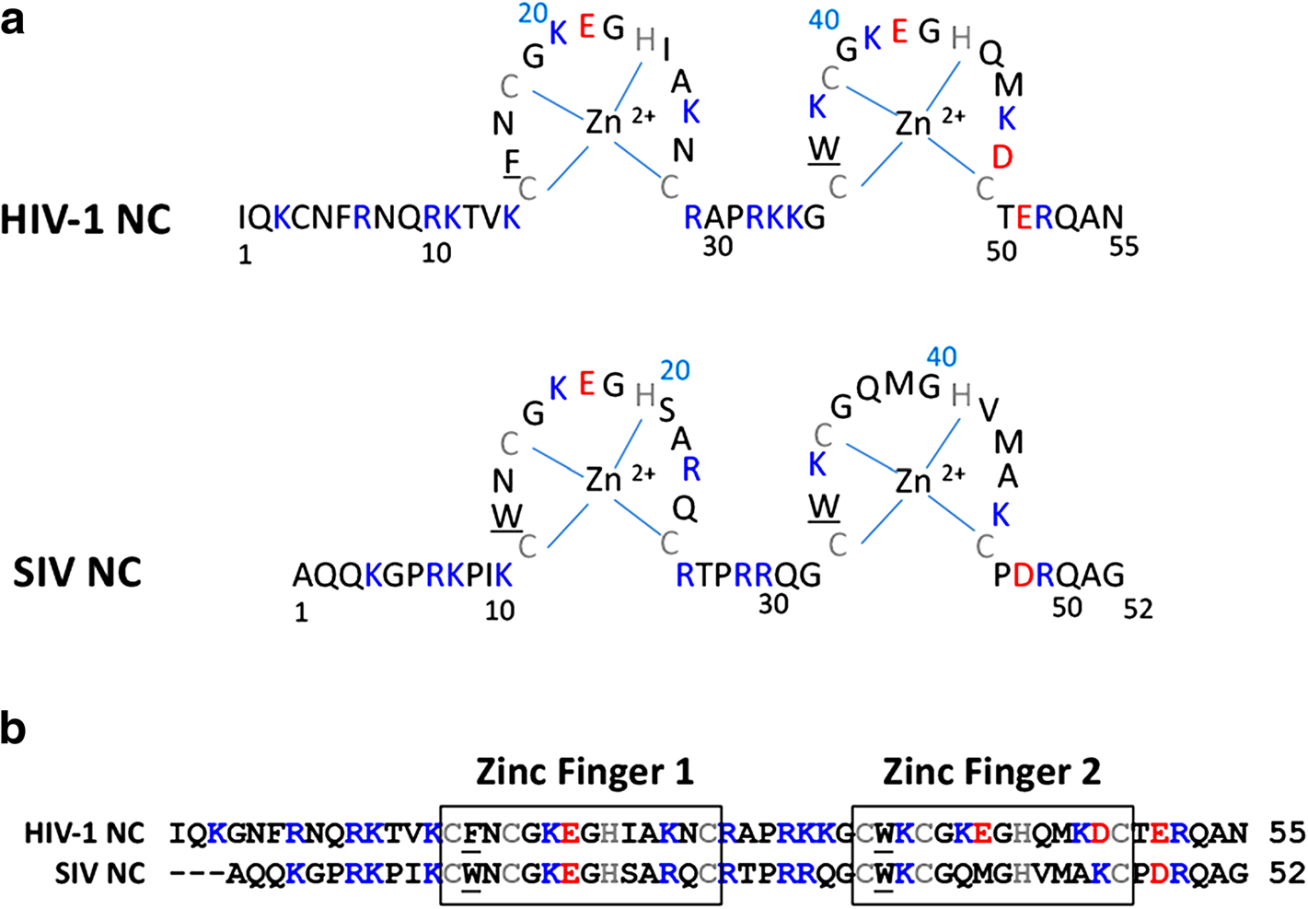
Sequence and structural features of HIV-1 and SIV NC proteins. **a** Schematic diagrams of NC proteins: HIV-1 NL4-3 NC and SIVmne NC. Basic residues are *colored blue*, acidic residues are *colored red*, the CCHC residues that coordinate the Zn^2+^ ions in the ZFs are *colored gray*, and the aromatic residue in each finger is underlined. The numbering is based on the sequence of the mature NC protein in each case. **b** Sequence alignment of HIV-1 and SIV NC proteins. *Coloring* and *underlining* are the same as in (**a**). The *boxes* indicate the sequence comprising each ZF

Like other retroviruses, SIV and HIV-1 have a nucleocapsid protein (NC), a small basic structural protein containing two zinc-binding domains, i.e., zinc fingers (ZFs), each with the invariant CCHC motif, connected by a short basic flexible peptide (Fig. 1a) [16–20]. NC is generated by viral protease (PR)-mediated cleavage of the Gag precursor protein during virus maturation [21–24]. For HIV-1, it has been shown that the NC domain in Gag is essential for specific recognition of the Psi packaging element in genomic RNA (gRNA) [25–31] and tRNA^Lys3^ primer placement on gRNA [32–34].

Retroviral NC proteins are nucleic acid (NA) chaperones, i.e., they remodel NA structures to facilitate formation of the most thermodynamically stable conformations [35] (reviewed in refs. [17–19, 36, 37]). This activity is critical for ensuring specific and efficient reverse transcription, including initiation [38, 39], as well as the minus- and plus-strand transfer reactions [18, 19, 37]. For example, in the minus-strand transfer step, NC facilitates annealing of the complementary repeat regions (R, r), which contain the highly structured transactivation response element (TAR) in gRNA and its minus-strand DNA complement, respectively [18, 19, 40].

Effective chaperone activity consists of three components: (1) NA aggregation, which is important for NA annealing (associated with the basic residues); (2) moderate helix destabilizing activity (associated with the ZFs); and (3) rapid on–off NA binding kinetics [41] (reviewed in refs. [18, 19]). In fact, the relatively weak NA chaperone activity of the HIV-1 Gag and NCp9 precursor proteins compared with that of mature NC can be attributed to their slow dissociation kinetics from bound NA [42–46]. Recent work on the molecular mechanism responsible for NC-facilitated duplex destabilization has focused on structural elements required for murine leukemia virus (MLV) gRNA dimerization [47], as well as destabilization of HIV-1 TAR DNA [48], TAR RNA [49, 50], and full-length (−) strong-stop DNA ((−) SSDNA), which contains all of r and the complement of the unique 5′ sequence (u5) [51]. These studies have emphasized the importance of ZF-dependent binding of specific G bases and contributions of unstable stem structures with mismatched bases, bulges, and loop regions.

Not surprisingly, given the simian origin of HIV-1, SIV NC shares 53% amino acid sequence identity with the HIV-1 protein (Fig. 1). Early work demonstrated that both SIV from *Macaca nemestrina* (SIVmne) and HIV-1 NCs had very similar NA binding properties in studies with model oligo- and polynuclotide substrates [52]. Moreover, the NMR solution structure of SIVl’hoest NC (residues 13–51) showed that the overall structures of SIV and HIV-1 NC are also very similar, despite several amino acid sequence differences in the ZFs and structural differences in the flexible linker [53]. The ability of SIV NC to coordinate Zn^2+^ is required for efficient replication in cell-based assays [54, 55], Gag processing [55, 56], proper core condensation and NC protein stability [56], as well as RNA packaging, although to a lesser extent than for MLV and HIV-1 [54, 55]. In contrast to HIV-1 NC, ZF2 of SIV NC appears to be slightly more important than ZF1 [55, 56]. Interestingly, compared with the HIV-1 protein, the chaperone activity of HIV-2 NC is not as robust, likely due, at least in part, to the shorter HIV-2 N-terminal basic region [57].

In this study, we provide an in-depth analysis of the chaperone activities of SIV and HIV-1 NCs in the context of biologically relevant reactions: the minus-strand transfer step in reverse transcription and selective binding to the Psi packaging element. Using a variety of biochemical and biophysical (e.g., single molecule DNA stretching and dynamic light scattering) approaches, we show that the slightly higher activity of HIV-1 NC in the annealing reaction in minus-strand transfer can be explained by the greater aggregation activity of the HIV-1 protein relative to SIV NC. Salt-titration assays show that both NC proteins have a similar balance of electrostatic and specific binding contacts with NAs and both can distinguish HIV-1 Psi RNA from non-Psi sequences. However, neither protein is capable of specific binding to the putative SIV RNA packaging signal tested here. Overall, while most of the NA binding and chaperone activities of HIV-1 and SIV NC are similar, our analysis reveals mechanistic differences that provide unique information regarding the replication strategies of HIV-1 and SIV.

## Results

### Comparison of SIV and HIV-1 NC proteins and predicted secondary structures of TAR and Psi RNAs

The sequences of the SIV and HIV-1 NC proteins are compared in Fig. 1. Both proteins are highly basic and have two ZF domains containing conserved CCHC motifs and aromatic residues. However, HIV-1 NC is more basic than SIV NC over a wide range of pH; at pH 7, for example, the estimated charge for HIV-1 is 11.2 and for SIV, 10.2. Additionally, ZF1 of SIV NC has a Trp residue, whereas the corresponding amino acid in HIV-1 NC is Phe. This difference is expected to be minor, since mutation of F16 to W has little effect on HIV-1 NC NA chaperone activity and intravirion reverse transcription and no effect on infectivity [58].

**Fig. 2:**
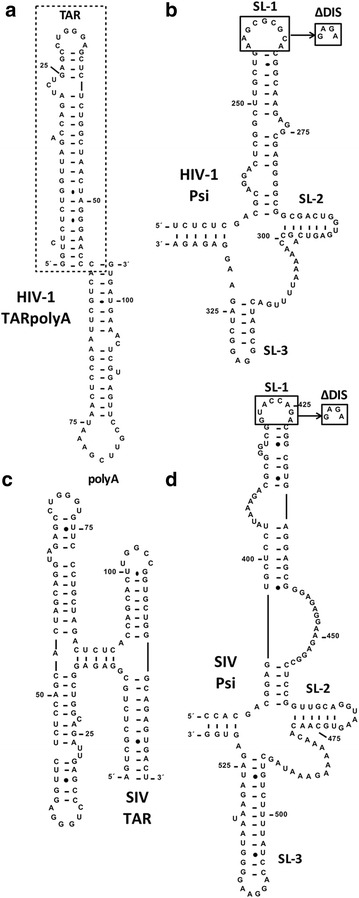
Sequence and mfold-predicted secondary structure of TAR and Psi RNA constructs used in this study. **a** HIV-1 TARpolyA. **b** HIV-1 Psi. **c** SIVmac TAR. **d** SIVmac Psi. In all cases, numbering refers to the nt position in gRNA. The *box* in (**a**) indicates HIV-1 TAR RNA. The *boxes* in (**b**, **d**) indicate the ∆DIS mutation, where DIS loop residues are replaced with a GNRA-type tetraloop (GAGA) to eliminate dimerization

During the course of virus replication, the HIV-1 NC protein interacts with structured RNA elements present at the 5′ end of the viral genome: the TAR stem-loop (SL), which is at the extreme 5′ end of R in gRNA (Fig. 2a) and is involved in the minus-strand transfer step of reverse transcription (reviewed in refs. [18, 19, 40]); and the Psi region, composed of three SLs, which contributes to the dimerization and packaging of gRNA and has been studied extensively [25, 59, 60]. The SL structures include SL1, which contains the dimerization initiation site (DIS) and two bulges, SL2 containing the major 5′ splice donor site; and SL3, which is important for packaging viral RNA (Fig. 2b). The earlier studies showed that while SL1-3 are all necessary for efficient gRNA encapsidation, SL1 and SL3 play a larger role than SL2. The RNA sequence that constitutes the SIV Psi element has not been studied to the same extent as that of HIV-1. The available information suggests that SIV and HIV-1 Psi share a similar secondary structure and that this region is also critical for SIV gRNA packaging [61–63] (Fig. 2d).

In the binding and small angle scattering (SAXS) experiments described below, we used an HIV-1 TARpolyA construct (Fig. 2a) to more closely match the longer and more complex SIV TAR structure (Fig. 2c) [63, 64]. Note that although the overall predicted secondary structures of HIV-1 and SIV Psi are similar (Fig. 2b, d, respectively), SIV Psi is 40 nucleotides (nt) longer than HIV-1 Psi, with significantly longer predicted SL1 and SL3 stem regions.

### Minus-strand annealing and strand transfer activities of SIV and HIV-1 NCs

**Fig. 3:**
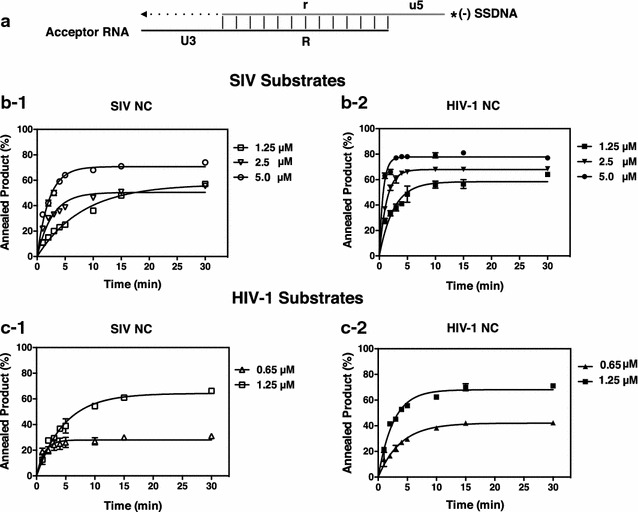
Kinetics of minus-strand annealing with SIV and HIV-1 substrates in the presence of SIV and HIV-1 NC proteins. **a** Reconstituted system used to assay minus-strand annealing and transfer. The *diagram* shows the acceptor RNA with a portion of U3 and the R sequence at the 3′ end of the viral genome annealed to (−) SSDNA with the complementary r sequence and a portion of u5, complementary to the U5 sequence. For the SIV substrates, the nt lengths of u5, R/r, and U3 sequences are as follows: u5, 20 nt; R/r, 176 nt; and U3, 52 nt. For the HIV-1 substrates, the lengths are: u5, 34 nt; R/r, 94 nt; and U3, 54 nt. The *asterisk* indicates that the (−) SSDNA is labeled at its 5′ end with ^32^P. Annealing of the complementary R regions is indicated by *vertical lines*. The U3 sequence serves as the template for RT-catalyzed extension of annealed (−) SSDNA. The final DNA transfer product is 248 nt (SIV) or 182 nt (HIV-1). The diagram is not drawn to scale. **b-1**, **b-2**, **c-1**, **c-2** Reactions were incubated with SIV (**b-1**, **b-2**) or HIV-1 substrates (**c-1**, **c-2**) and different concentrations of SIV NC or HIV-1 NC for 30 min at 37 °C and analyzed as described in “Methods” section. Representative gels can be found in Additional file 1: Fig. S1. The percent (%) annealed product was plotted against time of incubation. *Error bars* represent the standard deviation (SD) from three or more independent experiments

In view of the critical role of NC chaperone activity in reverse transcription [18, 19, 37], we investigated the activity of SIV and HIV-1 NCs in model systems that recapitulate the reactions required for minus-strand transfer (Fig. 3a). In the initial step, a DNA oligonucleotide representing (−) SSDNA (derived from sequences complementary to the 5′ end of the genome) is annealed to an RNA transcript representing the acceptor RNA (derived from sequences at the 3′ end of the genome) (Annealing). The annealed DNA is then extended by reverse transcriptase (RT) to give the transfer product (Minus-Strand Transfer). Note that in our systems, the minus-strand transfer assay includes the annealing step as well as DNA elongation.

To compare the annealing activities of the two NCs (Fig. 3), we evaluated reactions containing SIV substrates and either SIV NC (Fig. 3b-1) or HIV-1 NC (Fig. 3b-2), as well as reactions with HIV-1 substrates and SIV NC (Fig. 3c-1) or HIV-1 NC (Fig. 3c-2). Representative gels can be found in Additional file 1: Fig. S1. As the NC concentration was increased, the extent of annealing was also increased. For example, when 1.25 µM NC was used to measure SIV annealing, the percent annealed product was ~ 60% with both NCs, but at 5.0 µM NC, plateau values of almost 80% were reached (Fig. 3b-1, b-2). In general, at the 30 min end point, the percent annealed DNA was very similar for reactions with SIV or HIV-1 NC; in some cases the values obtained with HIV-1 NC (with either substrate set) were slightly higher than the values observed with SIV NC, but the difference was never greater than ~1.4-fold (e.g., reaction with HIV-1 substrates and NC at 0.65 µM: compare data in Fig. 3c-1 (31%) and Fig. 3c-2 (42%)).

In contrast, comparison of the rates of annealing showed small, but more significant differences between the activities of the two NCs (Table 1a). Thus, with the SIV substrates, the rate of annealing with HIV-1 NC was ~ 3-fold higher than the rate with SIV NC, whereas with the HIV-1 substrates, the difference was ~ 2-fold. SIV NC appeared to be somewhat more active with the HIV-1 substrates, but HIV-1 NC had the same activity in both systems.

**Table 1:** Rates of (a) annealing (k_obs_ values)^a^, (b) strand transfer (k_obs_ values)^b^

NC	SIV substrates (min^−1^)	HIV-1 substrates (min^−1^)
(a)		
SIV (1.25 µM)	0.12 ± 0.016^c^	0.20 ± 0.021
HIV-1 (1.25 µM)	0.39 ± 0.063	0.38 ± 0.035
(b)		
SIV (1.25 µM)	0.087 ± 0.004^c^	0.064 ± 0.004
HIV-1 (1.25 µM)	0.20 ± 0.01	0.17 ± 0.01

^a^Rates were determined by fitting the data from Fig. 3b-1, b-2, c-1, c-2 to a single exponential equation

^b^Rates were determined by fitting the data from Fig. 4b, c and Additional file 2: Figure S2 to a single exponential equation

^c^The error determinations represent the SD from three or more independent experiments

**Fig. 4:**
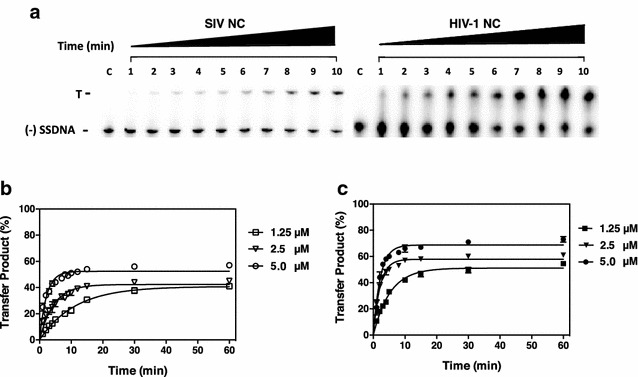
Kinetics of SIV minus-strand transfer in the presence of SIV and HIV-1 NC proteins. Reactions were incubated with the indicated concentrations of SIV or HIV-1 NC for 60 min at 37 °C and were analyzed as described in “Methods” section. **a** Representative gels showing DNA species present in reactions with 1.25 µM SIV or HIV-1 NC. The transfer product (T) and (−) SSDNA are indicated to the left of the gel image and these were the only two bands that appeared on the gel. Note that self-priming products [18, 19, 100, 114] were not formed under the conditions used for these assays. *Lane c* shows the migration of (−) SSDNA in the absence of other reactants. **b**, **c** The % strand transfer product formed was plotted against time of incubation for reactions with SIV NC (**b**) or HIV-1 NC (**c**). *Error bars* represent the SD from three or more independent experiments

Minus-strand transfer was tested next, initially in reactions with SIV substrates and either SIV or HIV-1 NC (Fig. 4). The gel images clearly show bands corresponding to the transfer product and (−) SSDNA (Fig. 4a). As was observed for annealing, the end point values for SIV minus-strand transfer were similar for reactions with SIV (Fig. 4b) or HIV-1 (Fig. 4c) NC. For example, at 1.25 µM NC, the percent strand transfer with SIV NC was 42% and with HIV-1 NC, it was 51%. At 5 µM, the plateau values were 57% (SIV NC) and 70% (HIV-1 NC). Again, there was a small, but more significant difference in the reaction rates with the two NCs. With 1.25 µM HIV-1 NC, the rate was 2.3-fold greater than with SIV NC at the same concentration. (Table 1b). Not surprisingly, minus-strand transfer with the HIV-1 substrates was slightly more efficient than with the SIV substrates, but in this case too, the end point values with 1.25 µM NC were very similar (51%, SIV NC; 64%, HIV-1 NC) (Additional file 2: Fig. S2). The rate of the reaction was 2.6-fold higher with HIV-1 NC (Table 1b).

### Single molecule DNA stretching experiments

**Fig. 5:**
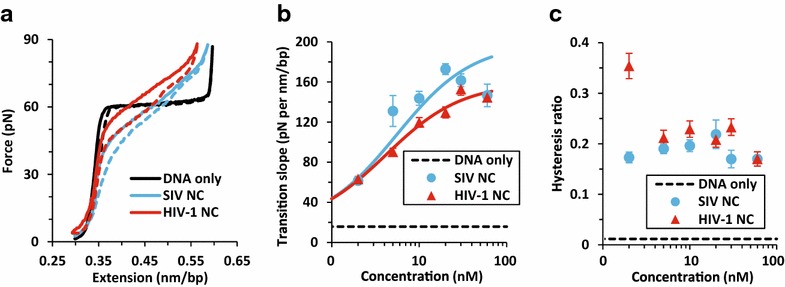
**a** Force-extension curves for dsDNA stretch (*solid lines*) and return (*dashed lines*) with no protein and in the presence of 30 nM SIV NC or HIV-1 NC. **b**, **c** Dependence of the measured transition slope (**b**) and hysteresis area ratio (**c**) on protein concentration (see Additional file 4) for HIV-1 NC and SIV NC. The *lines* in (**b**) are fits to a simple binding isotherm (Additional file 4: Eq. 6), revealing equilibrium dissociation constants *K*_d_ = 5.5 ± 0.4 nM for SIV NC and *K*_d_ = 4.2 ± 0.4 nM for HIV-1 NC. *Error bars* are standard errors for three or more measurements

To further understand differences between HIV-1 NC and SIV NC, we tested the force-extension (stretch and return) curves of single DNA molecules as a function of protein concentration for both proteins. As shown in Fig. 5a, the shapes and qualitative characteristics of the force-extension curves were very similar. To quantify these characteristics, we calculated the transition slope, which reflects the degree of intercalative binding by NC to DNA, and the hysteresis area ratio, which reflects the amount of strand separation, as a function of concentration [58, 65, 66] (Additional file 3: Fig. S3, Additional file 4).

The slope of the force-extension curve is measured near the midpoint of the transition by averaging over the extensions between 0.4 and 0.5 nm/base pair (bp). To determine the hysteresis area ratio, we find the linear combination of the worm-like chain (WLC) model, which describes double-stranded DNA (dsDNA) (Additional file 4: Eq. 1), and the freely-jointed chain (FJC), which describes single-stranded DNA (ssDNA) (Additional file 4: Eq. 2) that intersects the highest extension of the data (Additional file 4: Eqs. 3–4). This makes it possible to obtain a value for the hysteresis area ratio (Additional file 4: Eq. 5) that is independent of how far the DNA is stretched for a particular curve. The results of these quantitative analyses as a function of concentration are shown in Fig. 5b, c. The transition slope measurements suggest that SIV and HIV-1 NC have similar binding affinities in the nM range, as shown by their nearly identical equilibrium dissociation constants: *K*_d_ = 5.5 ± 0.4 and 4.2 ± 0.4 nM, respectively (Fig. 5b). The overall maximum transition slope is slightly higher for SIV NC, consistent with its slightly stronger intercalative binding relative to HIV-1 NC. Analysis of the hysteresis as a function of concentration also shows that for the lowest concentrations tested, HIV-1 NC binding resulted in a larger hysteresis area ratio compared to SIV NC. At all other concentrations tested, the proteins behaved in a very similar manner (Fig. 5c).

**Fig. 6:**
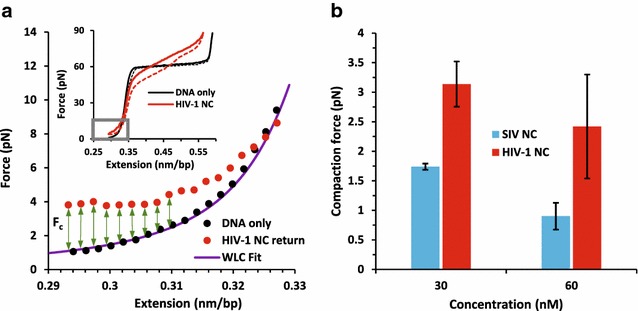
**a** Method for calculating the compaction force (F_c_) induced by protein-DNA interactions. Inset shows stretch (*solid lines*) and return (*dashed lines*) curves for dsDNA in the absence of protein and in the presence of near saturated (30 nM) HIV-1 NC protein. F_c_ is calculated in the low force-extension regime denoted within the *gray box* in the *inset* and magnified in the main figure. The DNA only extension curve is fit to the WLC model (Additional file 4: Eq. 1). The force difference (F_c_) between the return curve in the presence of high protein concentration and the DNA only stretching curve is averaged over measured extensions <0.31 nm/bp to obtain F_c_. **b** F_c_ for SIV NC and HIV-1 NC for concentrations of 30 nM and 60 nM. *Error bars* are standard errors for three or more measurements

The primary difference between HIV-1 NC and SIV NC, as observed in single molecule DNA stretching experiments, can be seen upon close examination of the force-extension curves at low forces and extensions (Fig. 6a). To stretch dsDNA at extensions below the dsDNA contour length of 0.34 nm/bp in the presence of protein, higher forces are needed relative to the “DNA only” sample. This additional force at low extensions is referred to as the DNA compaction force (F_c_). The magnitude of the F_c_ reflects the ability of the protein to attract dsDNA, which normally results in DNA aggregation in the absence of applied force [67, 68]. To quantify this compaction force, we used the method described in the legend to Fig. 6a. The results showed that the compaction force for HIV-1 NC is ~ 2-fold higher than that of SIV NC at both 30 nM and 60 nM concentrations (Fig. 6b). The additional compaction force for HIV-1 NC relative to that of SIV NC, averaged over both concentrations and weighted by uncertainty, is 1.3 ± 0.4 pN. This corresponds to a difference in compaction energy of 0.11 ± 0.03 k_B_T/bp. Thus, for a 10 kbp dsDNA molecule, similar to the length of the HIV-1 genome, the additional compaction energy for HIV-1 NC is 1100 ± 300 k_B_T, or 640 ± 170 kcal/mol, which is a very large energy difference for a molecular process.

### Analysis of HIV-1 and SIV NC NA aggregation properties by dynamic light scattering (DLS)

**Fig. 7:**
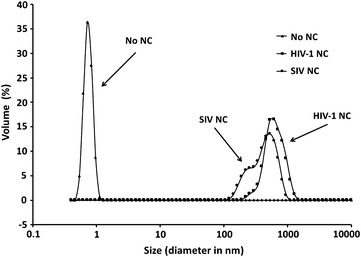
DLS measurements for HIV-1 and SIV NC proteins in the presence of SIV Psi RNA. The size distributions of NA aggregates formed in the presence of the indicated NC or a no NC control are shown. Each curve represents the average of at least three independent experiments

The ability of retroviral NC proteins to aggregate NAs is important for NC’s NA chaperone function, as well as for formation of the ribonucleoprotein complex containing gRNA that is located within the mature virion core [18, 69–72]. We used DLS to characterize the NA aggregate size generated by HIV-1 and SIV NCs in the presence of SIV Psi RNA (Fig. 7). In addition to the reactions with NC, a no NC control was included. The average size of the NA aggregate formed in the absence of NC was 0.74 ± 0.02 nm (n = 4) in diameter and ranged from 0.54 to 1.1 nm, consistent with the lack of aggregation under these conditions. In contrast, HIV-1 NC generated aggregates with a mean diameter of 642 ± 60 nm (n = 3) and a range from 164 to 1484 nm. These values agree with previous reports using different NA substrates [45, 69, 70]. Interestingly, the range of NA aggregates produced by SIV NC was found to be only 106 to 1106 nm, with an average size of 448 ± 65 nm (n = 5), which is smaller than the corresponding NA aggregates produced by HIV-1 NC. Taken together, these data suggest that HIV-1 NC is a more effective aggregating agent than SIV NC, consistent with the F_c_ measurements (Fig. 6b).

### RNA Binding properties of HIV-1 and SIV NC proteins

Retroviral NC proteins interact with NAs using both specific and non-specific modes of binding [27]. To evaluate the RNA binding properties of SIV and HIV-1 NCs, we examined the interaction of these proteins with four RNA constructs: HIV-1 TARpolyA; SIV TAR; HIV-1 Psi; and SIV Psi (Fig. 2). Since HIV-1 TARpolyA and SIV TAR sequences have been shown to be largely dispensable for selective gRNA packaging in HIV-1 and SIV virions, respectively, [25, 61, 62, 73, 74], we used these non-Psi RNAs to assay non-specific binding.

Fluorescence anisotropy (FA) salt-titration assays have previously been shown to be capable of distinguishing the relative contribution of specific vs. non-specific or electrostatic interactions for any given NC-RNA binding event [27, 75]. Briefly, the FA signal emitted by fluorescently-labeled RNA is measured at fixed protein and RNA concentrations, while the NaCl concentration is varied. As the salt concentration increases, less NC is able to bind RNA, resulting in a decrease in the FA signal. Thus, a protein-RNA complex that is more dependent on electrostatic interactions than specific contacts, dissociates at lower salt concentration relative to a complex that is characterized by specific non-electrostatic binding interactions. To quantify the results, the data are fit to an equation (see “Methods”), which yields the parameters *K*_*d*(1M)_ and *Z*_eff_. *K*_d(1M)_ represents the *K*_d_ of the protein-RNA interaction when all electrostatic contacts have been screened out and only specific ones remain (e.g., hydrogen bonding or aromatic stacking interactions). *Z*_eff_ represents the number of Na^+^ ions displaced from the RNA upon protein binding, which corresponds to the number of electrostatic contacts made between the protein and the RNA.

**Fig. 8:**
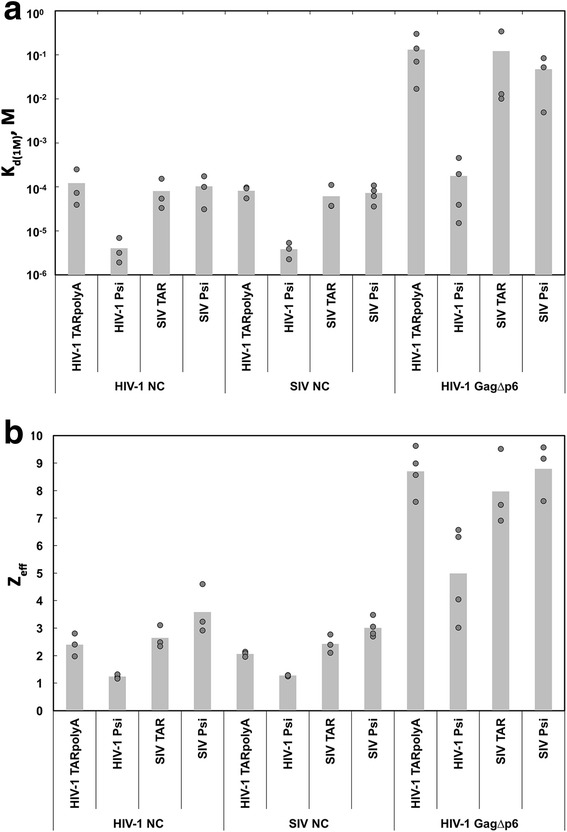
Plot of the parameters determined from measuring the interaction between HIV-1 NC, SIV NC, and HIV-1 Gag and the indicated HIV-1 and SIV RNAs as a function of salt concentration. The *dark gray circles* indicate the fitted **a** *K*_d(1M)_ (M = molarity) and **b** *Z*_eff_ parameters from each individual salt-titration experiment, while each *light gray bar* is the average of at least three independent trials

We performed FA salt-titration assays using the four SIV and HIV-1 RNAs shown in Fig. 2 and the corresponding NC proteins (Additional file 5: Fig. S4a–d). HIV-1 NC binding to HIV-1 TARpolyA and Psi substrates was characterized by *K*_d(1M)_ values equal to 1.2 × 10^−4^ M and 4.0 × 10^−6^ M, respectively, and *Z*_eff_ values equal to 2.4 and 1.2, respectively (Fig. 8; Table 2). The significant difference (~30-fold) between the *K*_d(1M)_ values for the HIV-1 NC-Psi and TARpolyA interactions is in general agreement with our previous report and shows that NC binds more specifically to Psi RNA relative to TARpolyA [27]. SIV NC binding to HIV-1 TARpolyA and Psi RNAs yielded *K*_d(1M)_ values of 8.2 × 10^−5^ M and 3.9 × 10^−6^ M, respectively, and *Z*_eff_ values of 2.1 and 1.3, respectively (Fig. 8; Table 2). These values are very similar to the values obtained with HIV-1 NC. Comparable binding affinities of HIV-1 and SIV NC to SL structures in HIV-1 Psi have also been reported as “unpublished results” in Ref. [52]. However, the current data also indicate that both proteins bind HIV-1 Psi RNA in a more specific, non-electrostatic manner than TARpolyA RNA.

**Table 2:** Binding parameters determined from FA salt-titration assays

RNA	HIV-1 NC		SIV NC		HIV-1 Gag∆p6	
	*K*_d(1M)_ (M)^a^	*Z*_eff_^b^	*K*_d(1M)_ (M)^a^	*Z*_eff_^b^	*K*_d(1M)_ (M)^a^	*Z*_eff_^b^
HIV-1 TARpolyA	(1.2 ± 1.2) × 10^−4^	2.4 ± 0.4	(8.2 ± 2.3) × 10^−5^	2.1 ± 0.1	(1.3 ± 0.1) × 10^−1^	8.7 ± 0.5
HIV-1 Psi	(4.0 ± 2.6) × 10^−6^	1.2 ± 0.1	(3.9 ± 1.5) × 10^−6^	1.3 ± 0.1	(1.8 ± 2.0) × 10^−4^	5.0 ± 2.0
SIV TAR	(8.1 ± 6.5) × 10^−5^	2.6 ± 0.4	(6.2 ± 4.3) × 10^−5^	2.4 ± 0.3	(1.2 ± 1.9) × 10^−1^	8.0 ± 1.4
SIV Psi	(1.0 ± 0.7) × 10^−4^	3.6 ± 0.9	(7.2 ± 3.1) × 10^−5^	3.0 ± 0.3	(4.7 ± 4.0) × 10^−2^	8.8 ± 1.0

All values represent the average of at least three trials with the associated standard deviation

^a^K_d(1M)_ is the affinity at 1 M NaCl, which represents the specific, non-electrostatic component of binding

^b^Z_eff_ is the number of Na^+^ ions released upon protein binding, and reflects the number of electrostatic contacts between protein and RNA

In contrast, HIV-1 NC was found to interact with SIV TAR and Psi RNAs with very similar *K*_d(1M)_ values of 8.1 × 10^−5^ M and 1.0 × 10^−4^ M, respectively, and *Z*_eff_ values of 2.6 and 3.6, respectively (Fig. 8; Table 2). SIV NC was also unable to effectively discriminate between SIV TAR and Psi RNA interactions with *K*_d(1M)_ values of 6.2 × 10^−5^ M and 7.2 × 10^−5^ M, respectively, and *Z*_eff_ values of 2.4 and 3.0, respectively. These results suggest that neither HIV-1 nor SIV NC interacts with the SIV Psi RNA construct used here with greater specificity than with a non-Psi sequence and also show that regardless of the RNA substrate tested, both HIV-1 and SIV NCs interacted with very similar *K*_d(1M)_ and *Z*_eff_ values (Fig. 8; Table 2).

### SIV RNA interactions with HIV-1 Gag

We next wanted to establish whether specific Psi RNA recognition in the SIV system requires a Gag polyprotein. In earlier work, it was demonstrated that HIV-1 Gag∆p6 binds to HIV-1 Psi RNA with even greater specificity than the NC domain alone [27]. It was therefore of interest to test HIV-1 Gag’s binding to SIV Psi versus TAR RNA (Fig. 8; Additional file 6: Fig. S5).

In accord with previous results [27], HIV-1 Gag clearly exhibited specific binding to HIV-1 Psi versus TARpolyA with *K*_d(1M)_ values of 1.8 × 10^−4^ M and 1.3 × 10^−1^ M, respectively (Fig. 8a; Table 2). Similarly, *Z*_eff_ values of 5.0 and 8.7 for binding to HIV-1 Psi and TARpolyA, respectively (Fig. 8b) were in good agreement with the values obtained in the earlier study [27]. In contrast, when we tested HIV-1 Gag with SIV TAR and Psi RNAs, the *K*_d(1M)_ and *Z*_eff_ values were very similar: *K*_d(1M)_ values of 1.2 × 10^−1^ M and 4.7 × 10^−2^ M, respectively, and *Z*_eff_ values of 8.0 and 8.8, respectively (Fig. 8; Table 2). Thus, as we found with the NC proteins, HIV-1 Gag was also unable to differentiate the SIV Psi RNA construct from the non-Psi TAR RNA.

### SAXS reveals overall shape of SIV Psi RNA

We considered the possibility that the NC proteins and HIV-1 Gag are not able to specifically bind SIV Psi RNA due to structural differences between HIV-1 and SIV Psi RNAs. The structure of the HIV-1 Psi construct used for the current work was previously characterized by SAXS [28] and we now applied this approach to SIV Psi. To ensure that the RNA was monomeric, the wild-type (WT) DIS loop was replaced with a GNRA-type GAGA tetraloop (∆DIS mutation, Fig. 2d). SIV Psi-∆DIS was purified by size exclusion chromatography (SEC) prior to analysis by SAXS. The SEC trace and subsequent analysis by electrophoresis in a native gel (performed concomitantly with SAXS data acquisition) confirmed that SIV Psi-∆DIS was predominantly monomeric (Additional file 7: Fig. S6a).

Details of the SAXS data analysis and results are given in Additional file 7: Fig. S6, Additional file 8: Fig. S7, Additional file 9: Table S1, and Additional file 10. The SAXS envelope generated for SIV Psi-∆DIS was compared to that of HIV-1 Psi-∆DIS (Additional file 8: Fig. S7). As previously reported, all stem-loops of HIV-1 Psi-∆DIS are solvent exposed and clearly discernable in the envelope with apparent co-axial stacking between SL1 and SL3 [28]. In contrast, the SIV Psi RNA appears more globular and there is no apparent co-axial stacking between the stem-loops. The SAXS data are therefore consistent with the conclusion that SIV Psi-∆DIS adopts an altered global fold relative to HIV-1 Psi-∆DIS.

## Discussion

The goal of the present study was to obtain a detailed comparison of the NA chaperone activities of SIV NC and the more extensively characterized HIV-1 protein. This issue is of great importance in view of the essential role of the NC protein in retrovirus replication [18–20, 76] and the widespread use of simian model systems for studies on HIV-1 pathogenesis, vaccine development, and drug resistance [77, 78], and more recently, the development of HIV-1 eradication and CURE strategies [79]. Here, we focus on two important events in the virus life cycle: the minus-strand transfer step in reverse transcription that is required for synthesis of a full-length copy of the viral RNA genome; and selective gRNA packaging directed by the Psi structural element. Differences in the global folds of putative SIV and HIV-1 Psi RNA sequences were uncovered in this study, although SIV and HIV-1 NC proteins exhibited similar behavior when interacting with each of these RNAs. Our results are consistent with the earlier NMR structural study of SIV NC [53], as well as with previous functional analysis [52], which also came to the conclusion that SIV NC is highly similar to the HIV-1 protein. However, we also demonstrated that despite an overall functional similarity, quantitative differences in NA aggregation and compaction capability distinguish the two proteins, which may be relevant to the infection process.

In our initial approach, we examined the minus-strand transfer reaction, since the rate-limiting step, i.e., annealing of the complementary R regions, is strongly dependent on NA chaperone activity to transiently destabilize the TAR structures and facilitate efficient NA binding. Assays of annealing or annealing plus DNA elongation (Figs. 3, 4; Additional file 1: Fig. S1, Additional file 2: Fig. S2) showed that the SIV NC-mediated reactions occur at a 2- to 3-fold slower rate than with HIV-1 NC (Table 1), although the extent of product formation after incubation for 30 or 60 min is only slightly elevated in the presence of HIV-1 NC. To understand the physical basis for this behavior, we performed single molecule DNA stretching and DLS experiments.

In single molecule stretching determinations, both HIV-1 and SIV NC showed very similar NA binding affinities and qualitative interactions with DNA, consistent with their very similar domain structure (Fig. 5). However, a more quantitative analysis revealed a significant difference in the compaction forces, F_c_s, generated by these two proteins at extensions less than the DNA contour length (Fig. 6). The F_c_s induced by SIV NC were 2-fold lower than those of HIV-1 NC, leading to a very large difference in the DNA compaction energy for the two proteins. Since the N-terminal domain is believed to be a primary determinant for HIV-1 NC’s aggregation properties [41], this lower F_c_ could be due to the shorter N-terminal domain of SIV NC, which has one less basic residue, relative to that of HIV-1 NC. In addition, the ZF linker domain of HIV-1 NC has a much higher charge density (5 basic residues) than SIV NC (3 basic residues), and this is also likely to contribute to the stronger aggregation ability of the HIV-1 protein. DLS measurements to assess NA aggregate size showed that SIV NC produces NA aggregates with a smaller average size and smaller size distribution than HIV-1 NC (Fig. 7), in excellent agreement with the single molecule stretching data. Thus, the observed difference in NA chaperone activity of HIV-1 NC relative to SIV NC, although modest, is likely due to stronger aggregation and electrostatic interaction properties of the HIV-1 protein. Moreover, single molecule DNA compaction energy measurements suggested that the differences between the NA interactions of HIV-1 NC and SIV NC may be amplified for NA chaperone functions involving longer NAs.

The smaller slope of the DNA stretching curves observed in the presence of HIV-1 NC compared to SIV NC (Fig. 5b), reflects less optimal intercalative binding to the DNA duplex. We hypothesize that this may be due to the presence of a Trp residue in ZF1 of SIV NC instead of the Phe present in HIV-1 NC, and that this subtle difference may lead to stronger stacking and intercalation. Interestingly, feline immunodeficiency virus (FIV) NC binding resulted in an even smaller transition slope than HIV-1 NC at all concentrations tested [66]. FIV NC also has one aromatic residue in each ZF, but the aromatic residue in ZF2 is located on the opposite side of the finger relative to that on SIV NC and HIV-1 NC. These data suggest that both SIV and HIV-1 NC intercalate more strongly than FIV NC, consistent with the non-optimal location of the aromatic amino acid in ZF2 of the FIV protein [66].

We also showed using a FA salt-titration binding assay that HIV-1 and SIV NCs interact with a very similar degree of electrostatic versus specific binding contacts, independent of the RNA examined. Importantly, like HIV-1 NC, SIV NC was capable of interacting with HIV Psi RNA using a more specific binding mode (i.e. lower *K*_d(1M)_) relative to a non-Psi RNA (HIV-1 TARpolyA) (Fig. 8). This finding is consistent with the previous observation that SIV proteins are capable of packaging and transducing HIV-1 gRNA [80], although a separate study reported that HIV-1 gRNA packaging by SIV GagPol occurred at a reduced efficiency and HIV-1 gRNA transfer to SIV target cells was not observed [81]. A recent study showed that both HIV-2 NC and HIV-2 Gag∆p6 preferentially bind HIV-2 Psi RNA [82]. However, the affinity of Gag for the Psi element is greater than that of NC, reflecting contributions from both the NC and MA domains in Gag. Interestingly, the mature HIV-2 MA protein also has NA chaperone activity, but unlike mature HIV-2 NC, is unable to distinguish Psi and non-Psi RNAs. Another recent report consistent with our results found that the dimerization properties of HIV-1 and SIV 5′ leader RNAs are determined by their DIS sequence and not by the identity of the NC protein (HIV-1 vs. SIV) used to induce dimerization [83]. This led the authors to conclude that the HIV-1 and SIV NC proteins are functionally equivalent in their ability to promote RNA dimerization.

In contrast to binding data with HIV-1 Psi, neither SIV NC nor HIV-1 NC interacted with the putative SIV Psi RNA in the more specific binding mode and the interaction profiles were not readily distinguishable from that of either HIV-1 TARpolyA or SIV TAR (Fig. 8; Table 2). HIV-1 Gag was also unable to discriminate between SIV Psi versus TAR RNAs, even though it bound HIV-1 Psi with high specificity. This result is surprising in light of reports showing that HIV-1 Gag/GagPol can package and propagate SIV gRNA [81, 84]. Taken together, these observations suggest that it may be the NA sequence that we have selected as “SIV Psi”, rather than an inability of SIV NC to make specific NA interactions, which is responsible for the lack of observed specificity.

It is important to note that the minimal Psi packaging element has not yet been unambiguously identified in HIV-1 [25, 30] or SIV [61, 62, 73, 74], as it has for other retroviruses such as MLV [85–87] or Rous sarcoma virus (RSV) [88–90]. While we derived both HIV and SIV Psi constructs used in this work from gRNA regions that have been shown in genetic experiments to be necessary for efficient genome packaging, additional sequences have been proposed to play a role in HIV-1 [59] and SIV [91] gRNA encapsidation. Despite the strong secondary structural homology between HIV-1 and SIV Psi [63], additional RNA sequences may be required for SIV Psi to fold into a well-defined packaging signal in vitro. Consistent with this possibility, the SAXS envelope of SIV Psi RNA appears less well defined and differs significantly from that of HIV-1 Psi. This is in contrast to MLV Psi, which has an overall fold that resembles that of HIV Psi [92]. Alternatively, we cannot rule out the possibility that SIV Gag is required for specific binding to the SIV Psi element. Indeed, we have previously shown that the MA domains of HIV-1 [27] and RSV [93] Gag enhance the specificity of binding to their cognate Psi RNAs.

In summary, the functional similarities between HIV-1 and SIV NC proteins are highlighted in the present work by their ability to interact specifically with HIV-1 Psi RNA and to effectively discriminate HIV-1 Psi versus non-Psi RNAs such as HIV-1 TARpolyA and SIV TAR, providing additional mechanistic insight into inter-species genomic RNA packaging. Nevertheless, despite the high structural and functional homology, our studies also clearly reveal subtle differences in the NA chaperone functions of HIV-1 and SIV NC proteins that can be explained by differences in their NA aggregation capabilities and DNA compaction energies.

## Conclusions

Based on biochemical assays and quantitative biophysical analysis, we demonstrated that despite a high degree of similarity between SIV and HIV-1 NC proteins, modest differences in their nucleic acid chaperone activities were observed, which reflect differences in DNA compaction energy and ability to aggregate NAs. In addition, we provided evidence for specific cross-species recognition of the HIV-1 Psi RNA packaging signal. Taken together, the vast similarities and only subtle differences observed in NC functional assays help to further validate SIV as a useful vehicle for development of new therapeutic strategies in the fight against the devastating consequences of AIDS.

## Methods

### Materials

DNA oligonucleotides and pIDTSMART vectors were purchased from Integrated DNA Technologies (IDT) (Coralville, IA, USA). [γ-^32^P]ATP was obtained from PerkinElmer (Shelton, CT, USA). HIV-1 RT was purchased from Worthington (Lakewood, NJ, USA). T4 polynucleotide kinase, proteinase K, SUPERaseIN, and Gel Loading Buffer II were bought from Life Technologies (Foster City, CA, USA). E271 loading dye base was obtained from AMRESCO LLC (Solon, OH, USA). The Ambion MEGAshortscript T7 kit was purchased from Life Technologies. The sequences of the HIV-1 acceptor RNA and (−) SSDNA, as well as the TAR and Psi RNAs were derived from HIV-1 NL4-3 (GenBank Accession no. AF324493) [94]. The corresponding SIV NAs were derived from SIVmac239 (GenBank Accession no. M33262) [12, 13], which was obtained from Dr. Ronald Desrosiers through the AIDS Reagent Program, Division of AIDS, NIAID, NIH.

### Recombinant NC and Gag proteins

HIV-1 recombinant NC proteins were expressed in *E. coli* BL21 (DE3) cells and purified as described previously [95, 96]. Essentially the same procedures were used to prepare the SIV NC proteins. Briefly, the DNA regions encoding the 52-amino-acid sequences from SIVmne (Genbank accession no. M32741) [97, 98] or SIVmac239 [12, 13] were cloned into the pET32a expression vector (Novagen, Inc., Madison, WI, USA), expressed in *E. coli*, cleaved from the thioredoxin fusion partner, and purified as described [95, 96]. Note that SIVmne and SIVmac239 NC proteins are identical except for the amino acids at positions 27, 38, and 40: the Mne residues are T27, Q38, and G40, respectively, whereas the Mac239 residues are A27, K38, and D40, respectively. In addition, there is functional identity at position 4, with K for Mne and R for Mac239. The experiments presented here were performed with SIVmne NC. SIVmac239 NC was used for some of the initial minus-strand annealing and strand transfer experiments; however, the results obtained with either SIV NC were the same within uncertainty (data not shown). The charge of each NC protein over a range of pH was calculated using the protein calculator at http://protcalc.sourceforge.net/. The HIV-1 Gag protein lacking the p6 domain (HIV-1 Gag∆p6, also referred to simply as “Gag”) was purified as previously described [27, 99]. The concentration of NC in solution was determined by measuring the absorbance at 280 nm using the extinction coefficients 5680 and 11,560 M^−1^ cm^−1^ for HIV-1 and SIV NCs, respectively, and for Gag, using the extinction coefficient 63,090 M^−1^ cm^−1^.

### Synthesis of viral RNA transcripts

The HIV-1 acceptor RNA (RNA 148) was prepared as described previously [100, 101]. The sequence of the SIV acceptor RNA (RNA 228) consisted of the 52 3′ terminal nt in the unique 3′ region (U3) (beginning at nt 10,184) to the last nt in R (nt 10,411). It was prepared by performing a PCR reaction using the SIV p239SpE3′ plasmid (GenBank Accession no. M33262) obtained from Dr. Ronald Desrosiers through the AIDS Reagent Program, Division of AIDS, NIAID, NIH [12, 13]. The dsDNA product was run on a 2.5% agarose gel and then gel purified prior to transcription with T7 RNA polymerase using the MEGAshortscript T7 kit. The SIV acceptor RNA product was subjected to electrophoresis in a 6% polyacrylamide denaturing gel and then gel purified prior to use in the minus-strand annealing and strand transfer assays.

For the salt-titration and SAXS experiments, DNA template sequences encoding the T7 RNA polymerase promoter sequence followed by the sequences for HIV-1 viral RNAs (TARpolyA and Psi-WT) were prepared as described [27]. DNA template sequences encoding the SIV RNAs (TAR and Psi-WT) cloned into pIDTSMART vectors were obtained from IDT. The HIV-1 and SIV Psi variants with DIS mutated to a GNRA tetraloop sequence (∆DIS) were generated from the Psi-WT plasmids using the QuikChange Lightning Site-Directed Mutagenesis kit (Agilent Technologies, Santa Clara, CA, USA). RNAs were prepared by in vitro transcription and purified as previously described [102]. Additional non-native G residues were added to all RNAs to facilitate efficient T7-mediated in vitro transcription: HIV-1 TARpolyA contains one additional G residue, HIV-1 Psi contains two additional G residues, SIV TAR contains two additional G residues, and SIV Psi contains two additional G residues. Purified RNAs were fluorescently labeled at their 3´ ends with fluorescein-5-thiosemicarbazide (Invitrogen, Carlsbad, CA, USA) as described [103, 104]. The RNA concentrations in solution were determined by measuring the absorbance at 260 nm, using the following extinction coefficients: HIV-1 TARpolyA, 935,693 M^−1^ cm^−1^; HIV-1 Psi-WT, 973,073 M^−1^ cm^−1^; HIV-1 Psi-∆DIS, 926,348 M^−1^ cm^−1^; SIV TAR, 1,113,248 M^−1^ cm^−1^; SIV Psi-WT, 1,318,838 M^−1^ cm^−1^; and SIV Psi-∆DIS, 1,281,458 M^−1^ cm^−1^. The extent of labeling with fluorescein was determined by measuring the absorbance at 495 nm and ε_495_ = 85,000 M^−1^ cm^−1^.

### Minus-strand annealing assay

A 196-nt DNA (DNA 196) (SIV) or a 128-nt DNA (DNA 128) (HIV-1) representing (−) SSDNA (0.2 pmol), labeled at its 5′ end with ^32^P [105, 106], was incubated at 37 °C with 0.4 pmol of acceptor RNA (RNA 228, SIV; RNA 148, HIV-1) in buffer containing 50 mM Tris–HCl (pH 8.0) and 75 mM KCl and the indicated concentrations of SIV or HIV-1 NC (final volume, 20 µl). Each substrate was tested with the same concentrations of SIV and HIV-1 NC. However, higher concentrations of both NCs were used for the SIV substrates, which were significantly longer than the HIV-1 substrates. The standard reaction was scaled up as needed and 15-µl portions were removed at intervals between 1 and 30 min. Reactions were terminated by addition of sodium dodecyl sulfate to a final concentration of 1% (vol/vol). The mixtures were placed on ice for 5 min and then extracted once with phenol/chloroform. Four µl of loading dye mix containing 12.5% glycerol (vol/vol) and 1x E271 loading dye base were added to 10 µl of the aqueous layer and an 8-µl portion was loaded onto a native 6% polyacrylamide gel prepared with a 4% stacking gel. Analysis of the gel data and calculation of the percent (%) annealed DNA were performed as described previously [107]. Note that to obtain efficient annealing and minus-strand transfer in the SIV system, the ratio of acceptor RNA to (−) SSDNA normally used in our HIV-1 system [100, 101] was increased from 1:1 to 2:1. For comparison, identical conditions were used for the HIV-1 system.

### Minus-strand transfer assay

Reaction mixtures containing reaction buffer (50 mM Tris–HCl (pH 8.0), 75 mM KCl, 1 mM dithiothreitol [DTT]), 0.2 pmol (−) SSDNA (DNA 196, SIV; DNA 128, HIV-1) labeled at its 5′ end with ^32^P, 0.4 pmol acceptor RNA (RNA 228, SIV; RNA 148, HIV-1), and SIV or HIV-1 NC as specified, were incubated for 5 min at 37 °C. HIV-1 RT (1 pmol) and 0.5 units SUPERaseIN were then added and the entire mixture was incubated for another 5 min at 37 °C. Reactions (final volume, 20 µl) were initiated by addition of 100 μM each of the four dNTPs, and 1 mM MgCl_2_. The standard reaction was scaled up as needed. Incubation was at 37 °C and 10-µl portions of the reaction mixture were removed at the indicated times. Reactions were terminated by addition of 4 µl of Gel Loading Buffer II. Polyacrylamide gel electrophoresis in 6% denaturing gels and PhosphorImager analysis were performed as described previously [107]. The % strand transfer product formed was calculated by dividing the amount of transfer product by the total signal in the gel lane and multiplying by 100 [108].

### Single molecule DNA stretching experiments

A biotinylated bacteriophage λ DNA molecule was tethered in between two streptavidin-coated polystyrene beads, torsionally unconstrained by its opposite ends. One bead was held in an optical trap, while the other was immobilized on a micropipette tip attached to a flow cell placed on a translational piezoelectric stage [109]. By gradually moving the fixed bead while recording the extension and the force exerted on the single DNA molecule, the force-extension profile of a dsDNA in the absence of protein was obtained. The buffer surrounding the DNA molecule was then exchanged for a solution of fixed HIV-1 or SIV NC protein concentration to obtain the force–extension curves in the presence of protein at a 100 nm/s pulling rate. The experiments were conducted in 10 mM 4-(2-hydroxyethyl)-1-piperazineethanesulfonic acid (HEPES), 50 mM Na^+^ buffer solution at pH 7.5.

### DLS measurements

DLS experiments were performed using 100 nM SIV Psi RNA in buffer containing 50 mM HEPES (pH 7.5), 5 mM DTT, 1.3 mM MgCl_2_, and 20 mM NaCl buffer. NC (1.2 µM) was added to the reaction mix and incubated at room temperature for 30 min prior to DLS measurement on a Zetasizer Nano-ZS instrument (Malvern Instruments Ltd, Malvern, Worcestershire, UK). Data were analyzed using the Dispersion Technology Software provided by the manufacturer, and sizes were plotted as volume percent versus particle size. The average size of the aggregate population produced was calculated by taking the product of the aggregate volume at each particle size sampled and averaging over the total volume of the population. The average diameter was calculated as the mean of 3–5 measurements with the standard error indicated.

### FA salt-titration binding assays

The salt-titration binding assays were performed essentially as previously described [27, 75]. Briefly, a fixed concentration of either HIV-1 or SIV NC (400 nM) was incubated with refolded RNA (10 nM) in increasing NaCl concentrations (30–750 mM) together with 20 mM HEPES (pH 7.5), 20 µM Tris-(2-carboxyethyl)-phosphine, 5 mM 2-mercaptoethanol, and 1 mM MgCl_2_. RNAs were refolded in 50 mM HEPES (pH 7.5) by heating at 80 °C for 2 min and then at 60 °C for 2 min, followed by addition of 10 mM MgCl_2_ and incubation on ice for at least 30 min. The reactions were incubated at room temperature in the dark for 30 min and then FA was measured using a SpectraMax M5 plate reader (Molecular Devices, Sunnyvale, CA, USA). Gag salt-titration assays were performed using the same protocol, except that 20 nM RNA was used and the reaction buffer also contained 2 mM Tris–HCl, pH 7.4. To correct for the effect of increasing NaCl on RNA anisotropy independent of protein binding, separate salt-titration assays of the RNA in the absence of protein were carried out with every trial. The no protein control values were then subtracted from the data obtained for protein-containing reactions. The corrected data were then analyzed as described [27, 75]. Briefly, the dissociation constant *K*_d_ varies as a function of Na^+^ ion concentration as follows:

1 $$ K_{\text{d}} = K_{{{\text{d}}(1{\text{M}})}} \cdot \, \left[ {\text{Na}} \right]^{\text{Zeff}} , $$

In Eq. 1, *K*_d(1M)_ is the dissociation constant of the RNA–protein interaction at 1 M NaCl when all electrostatic charges have been screened out, thereby reflecting the strength of the non-electrostatic binding contacts. *Z*_eff_ represents the number of electrostatic contacts involved in the interaction. Substituting Eq. 1 into the binding isotherm as previously described [27] allows determination of the two parameters, *K*_d(1M)_ and *Z*_eff_.

### Preparation of RNA for SAXS analysis

SIV Psi-∆DIS RNA (450 µg) was refolded as described above, except that an additional step of incubation at 37 °C for 5 min was added between the addition of MgCl_2_ and incubation on ice. The folded RNA was then purified via SEC on a 24-ml Superdex 200 10/300 GL Increase column (GE Healthcare, Little Chalfont, Buckinghamshire, UK) in running buffer containing 150 mM NaCl, 50 mM HEPES (pH 7.4), 1 mM MgCl_2_, and 3% glycerol (wt/vol) at a flow rate of 1 ml/min. Peak fractions containing the desired RNA were pooled and concentrated to 70–90 µl using an Amicon 0.5-ml 10 K molecular weight cutoff spin concentrator (EMD Millipore, Bellerica, MA, USA). Sample concentrations ranged from 3.0 to 3.7 µg/µl. The SEC running buffer was used to serially dilute the RNAs, yielding three sample concentration ranges (3.0–3.7, 1.5–1.9, and 0.75–0.93 µg/µl). An aliquot of the SEC buffer used to purify the RNAs was saved for use in SAXS for buffer subtraction.

### SAXS data acquisition and analysis

Samples were shipped in 96-well plates (Axygen Scientific, Union City, CA, USA) at 4 °C to the 12.3.1 SIBYLS beamline at the Advanced Light Source (Lawrence Berkeley National Lab, Berkeley, CA, USA) [110, 111]. Scattering data were acquired and buffer subtraction was performed by the SIBYLS beamline staff as described [111, 112]. Subsequent data analysis and ab initio envelope generation were performed largely as previously described [28]. Briefly, the SAXS data collected at different exposure times for a given concentration of sample were examined separately and exposures with clear evidence of radiation damage were discarded. High quality exposures for each RNA concentration dilution were merged and then analyzed using Guinier analysis [110] to calculate the radius of gyration (R_g_) and the extrapolated scattering intensity at zero scattering angle (I_0_) using the program PRIMUS [113]. Kratky analysis [110] was also performed for each RNA concentration dilution to confirm the extent of folding. If the R_g_ was found to increase upon increasing RNA concentration (indicative of concentration-dependent effects) or if the Kratky plots suggested that the RNA was not well folded, the data were not analyzed further. If samples passed these quality control analyses, then the data sets from the three concentration dilutions were scaled and merged into a single curve. The inter-electron P(r) functions were calculated using the program GNOM [113]. The maximum inter-electron distance (D_max_) was varied until the P(r) decayed smoothly to zero and the experimental data fit well. The D_max_ was increased by 2 Å increments up to 15 Å above the D_max_ initially selected by the GNOM program. Then for each of these D_max_ values, five ab initio envelopes were generated in fast mode with no symmetry restraints imposed using the ATSAS suite of programs as described [28, 113]. The average χ^2^ fit of the five envelopes to the experimental data was determined and the D_max_ condition that gave the best fit was chosen for further analysis. Using this D_max_, 20 ab initio envelopes were generated using the same protocol as described above, and the χ^2^ fits and reproducibility (NSD) values were calculated. These 20 envelopes were averaged into one envelope, which was then packed with at least 20,000 “dummy atoms” and used as the starting point for an additional 24 ab initio envelope calculations, generated in jagged mode with no symmetry restraints imposed. These envelopes were averaged to generate the final envelope and their χ^2^ fits and NSD values were determined.

## Additional files

**Additional file 1: Fig. S1.** Kinetics of minus-strand annealing with the SIV substrate in the presence of SIV or HIV-1 NC. Representative gels show DNA species present in reactions with the SIV substrates and 1.25 µM SIV or HIV-1 NC. Aliquots were removed from each reaction for gel analysis at the following times (min): 1; 2; 3; 4; 5; 10; 15; 30 (*lanes 1*–*8*, respectively). *Lane C* shows the migration of (−) SSDNA in the absence of other reactants. The annealed product and (−) SSDNA positions are indicated to the left of the gel image. Calculation of % annealing was based on the signal derived from the bands corresponding to the annealed product and (−) SSDNA. The two bands at the bottom of the gel may be small DNA oligonucleotides that were not completely removed in the purification procedure, as they are also present in the control (*lane C*).

**Additional file 2: Fig. S2.** Kinetics of HIV-1 minus-strand transfer in the presence of 1.25 µM SIV or HIV-1 NC. The % transfer product formed was plotted against time of incubation.

**Additional file 3: Fig. S3.** Method for calculating the transition slope and hysteresis area ratio. The *solid* and *dashed blue lines* represent the dsDNA stretch and return curves respectively, in the presence of protein. The *gold* and *pink lines* are theoretical models for dsDNA (WLC) and the ssDNA (FJC), respectively (Additional file 4). The *solid red line* is the WLC-FJC linear combination that intersects the highest force-extension data point (b_max_, F_max_), where b_max_ is the maximum extension reached and F_max_ is the maximum force reached. The slope of the linear least square fit (*dashed dark blue*) that describes the force-extension data between 0.4 and 0.5 nm/bp on the dsDNA stretch is defined to be the transition slope. The ratio of the area between the dsDNA stretch-return curves in the presence of protein (*filled yellow*) and the area between the stretch and the WLC-FJC linear combination (*yellow* + *green*) curves is defined as the hysteresis area ratio (Additional file 4: Supplementary Eqs. 1–5).

**Additional file 4.** Single molecule methods.

**Additional file 5: Fig. S4.** Salt-titration binding curves for the interaction of HIV-1 NC and SIV NC with HIV-1 and SIV RNAs. **a** HIV-1 TARpolyA. **b** HIV-1 Psi. **c** SIV TAR. **d** SIV Psi. Each curve represents the average of at least three independent experiments and the *error bars* are the standard deviation of the mean.

**Additional file 6: Fig. S5**. Salt-titration binding curves for the interaction of HIV-1 Gag∆p6 with HIV-1 Psi, SIV TAR, and SIV Psi. Each curve represents the average of at least three independent experiments and the *error bars* are the standard deviation of the mean.

**Additional file 7: Fig. S6.** Purification and SAXS curve analysis for SIV Psi-∆DIS RNA. **a** SEC trace of WT (*red*) and ∆DIS (*black*) SIV Psi RNAs reveals that mutation of the DIS loop is required to yield the monomeric species. Monomeric SIV Psi RNA was purified using this SEC protocol immediately prior to SAXS data collection. The *inset* shows analysis of the SEC-purified SIV Psi-∆DIS RNA by native polyacrylamide gel electrophoresis run concurrently with SAXS data collection of the same sample, confirming that the sample used in the SAXS experiment was a homogenous monomer. SIV Psi-WT was run as a control on the same gel, showing that it is predominately a dimer. **b** The Guinier plot of the low scattering angle (Å^−2^) region of the SAXS data fits well to a linear regression (*red line*) with low residuals (*green line*), indicating that the SIV Psi-∆DIS sample is not aggregated. **c** The Kratky plot shows a downward trend as momentum transfer/scattering angle increases, indicating that the RNA is nonglobular but well folded. The *blue line* represents the Kratky transformation of the scattering intensity and the *gray bars* represent the corresponding error in the intensity measurement. **d** The P(r) distribution plot indicates that the majority of electron pair distances lie between 25 and 65 Å for this RNA, and that the largest electron pair distance (i.e. D_max_) lies at 188 Å.

**Additional file 8: Fig. S7.** SAXS data obtained for SIV Psi-∆DIS RNA (146 nt) and comparison to HIV-1 Psi-∆DIS (105 nt). **a** Plot of intensity versus momentum transfer for SIV Psi-∆DIS RNA. The *open circles* indicate every fifth data point from the experimental SAXS curve and the *black line* represents the back-calculated scattering curve of the ab initio envelope. The χ^2^ fit between the experimental and back-calculated ab initio envelope scattering curves is reported. **b** Comparison of the SIV Psi-∆DIS and HIV-1 Psi-∆DIS envelopes showing the confirmed SL structures of the RNAs [28]. The two envelopes were superimposed using the SUPCOMB program [113] and the NSD value for the comparison is indicated.

**Additional file 9: Table S1.** Statistics determined from analysis of the SAXS scattering curves and construction of ab initio envelopes using DAMMIN.

**Additional file 10.** SAXS data analysis and results.

## Abbreviations

HIV-1: human immunodeficiency virus type 1
HIV-2: human immunodeficiency virus type 2
SIV: simian immunodeficiency virus
SIVcpz: SIV from chimpanzees
SIVsm: SIV from sooty mangabeys
SIVmac: SIV from macaques
NC: nucleocapsid protein
ZF: zinc finger
PR: protease
gRNA: genomic RNA
NA: nucleic acid
R, r: complementary repeat regions
TAR: transactivation response element
MLV: murine leukemia virus
(−) SSDNA: (−) strong-stop DNA
u5: complement of unique 5′ sequence
SIVmne: SIV from *Macaca nemestrina*
SL: stem-loop
DIS: dimerization initiation site
SAXS: small angle X-ray scattering
nt: nucleotide
RT: reverse transcriptase
bp: base pair
WLC: worm-like chain
dsDNA: double-stranded DNA
FJC: freely-jointed chain
ssDNA: single-stranded DNA
F_c_: DNA compaction force
DLS: dynamic light scattering
FA: fluorescence anisotropy
WT: wild-type
SEC: size exclusion chromatography
FIV: feline immunodeficiency virus
RSV: Rous sarcoma virus
U3: unique 3′ region
DTT: dithiothreitol
HEPES: 4-(2-hydroxyethyl)-1-piperazineethanesulfonic acid
R_g_: radius of gyration
P(r): pair distance distribution function
D_max_: maximum electron pair distance
NSD: normalized spatial discrepancy
