# Supplementary figures and images for: Mechanistic differences between HIV-1 and SIV nucleocapsid proteins and cross-species HIV-1 genomic RNA recognition
Source: Retrovirology. 2016 Dec 29;13:89. doi: 10.1186/s12977-016-0322-5 (PMC5198506; doi:10.1186/s12977-016-0322-5)

**Figure S1**

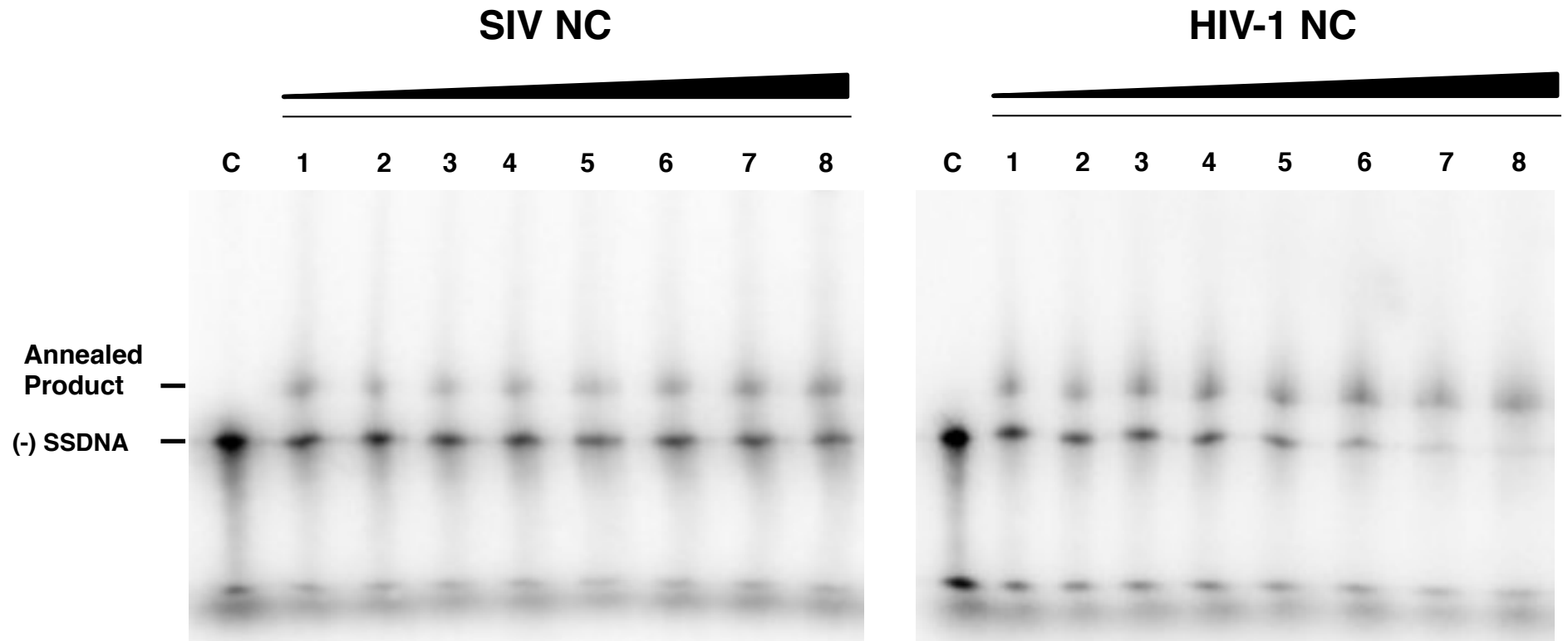

Supplement: Supplementary file 1 — Additional file 1: Fig. S1. Kinetics of minus-strand annealing with the SIV substrate in the presence of SIV or HIV-1 NC. Representative gels show DNA species present in reactions with the SIV substrates and 1.25 µM SIV or HIV-1 NC. Aliquots were removed from each reaction for gel analysis at the following times (min): 1; 2; 3; 4; 5; 10; 15; 30 (lanes 1–8, respectively). Lane C shows the migration of (−) SSDNA in the absence of other reactants. The annealed product and (−) SSDNA positions are indicated to the left of the gel image. Calculation of % annealing was based on the signal derived from the bands corresponding to the annealed product and (−) SSDNA. The two bands at the bottom of the gel may be small DNA oligonucleotides that were not completely removed in the purification procedure, as they are also present in the control (lane C). [file 12977_2016_322_MOESM1_ESM.pdf]

**Figure S2**

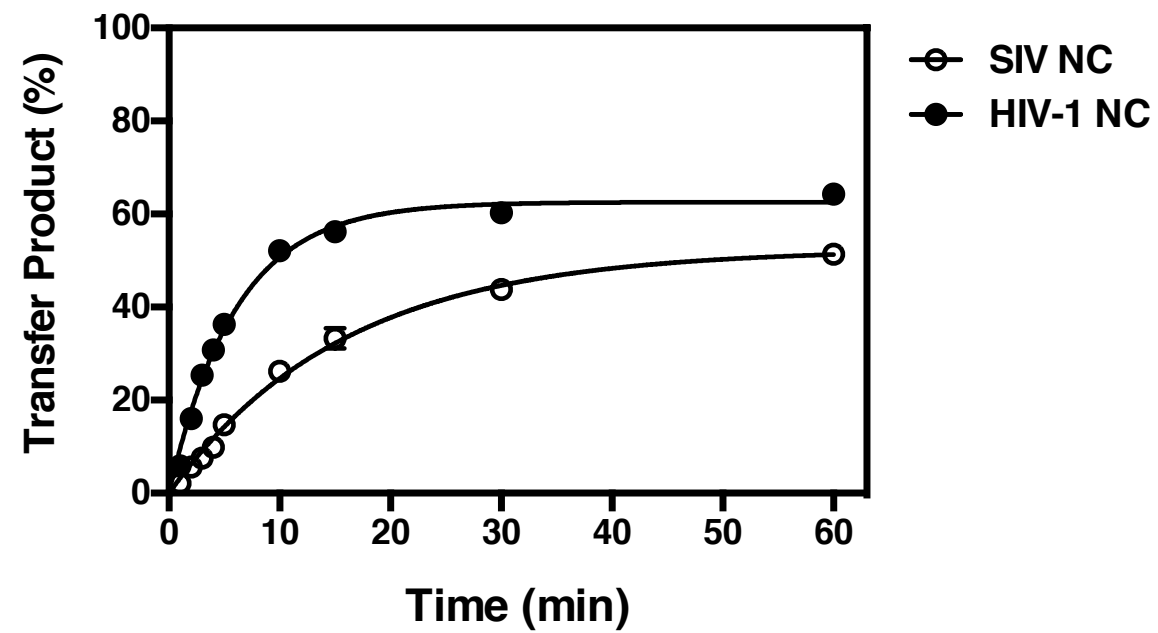

Supplement: Supplementary file 2 — Additional file 2: Fig. S2. Kinetics of HIV-1 minus-strand transfer in the presence of 1.25 µM SIV or HIV-1 NC. The % transfer product formed was plotted against time of incubation. [file 12977_2016_322_MOESM2_ESM.pdf]

Figure S3

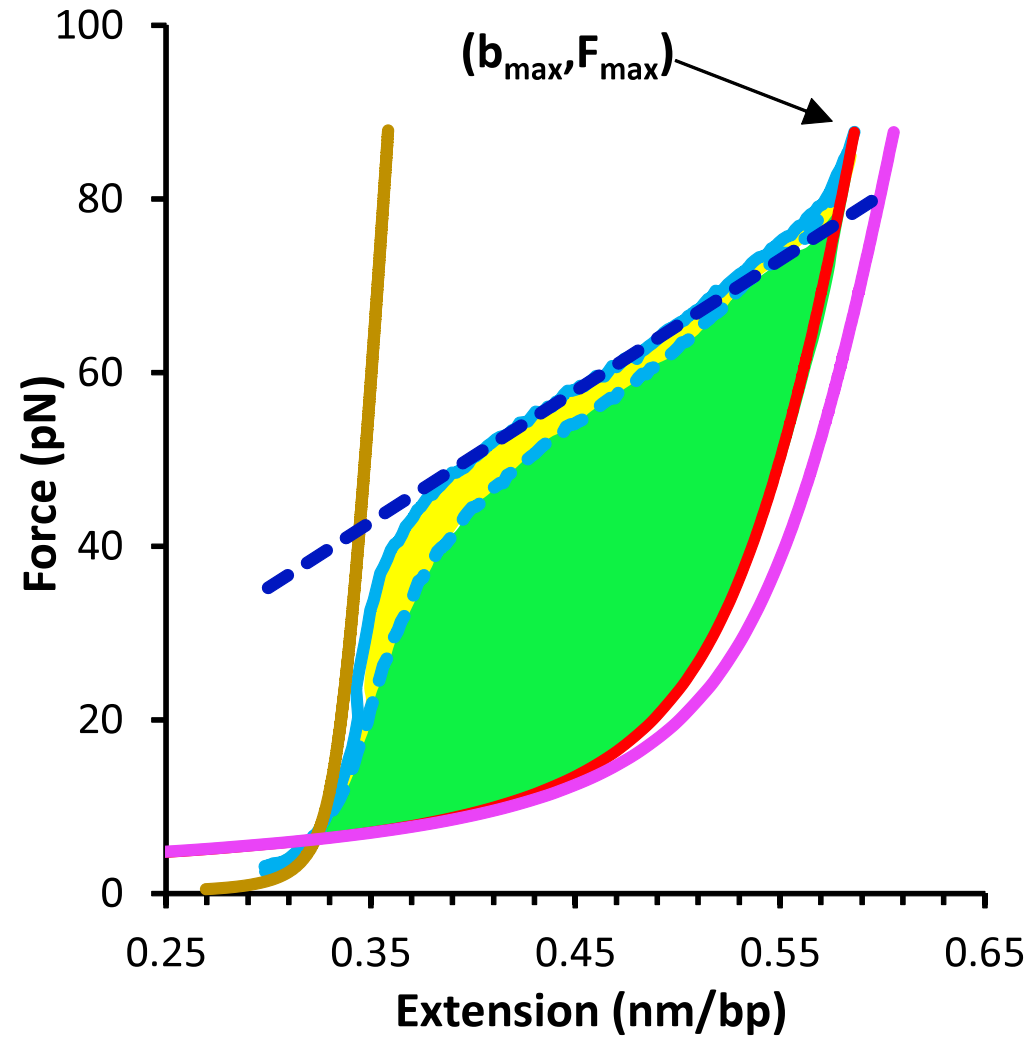

Supplement: Supplementary file 3 — Additional file 3: Fig. S3. Method for calculating the transition slope and hysteresis area ratio. The solid and dashed blue lines represent the dsDNA stretch and return curves respectively, in the presence of protein. The gold and pink lines are theoretical models for dsDNA (WLC) and the ssDNA (FJC), respectively (Additional file 4). The solid red line is the WLC-FJC linear combination that intersects the highest force-extension data point (bmax, Fmax), where bmax is the maximum extension reached and Fmax is the maximum force reached. The slope of the linear least square fit (dashed dark blue) that describes the force-extension data between 0.4 and 0.5 nm/bp on the dsDNA stretch is defined to be the transition slope. The ratio of the area between the dsDNA stretch-return curves in the presence of protein (filled yellow) and the area between the stretch and the WLC-FJC linear combination (yellow + green) curves is defined as the hysteresis area ratio (Additional file 4: Supplementary Eqs. 1–5). [file 12977_2016_322_MOESM3_ESM.pdf]

# Figure S4

**a**

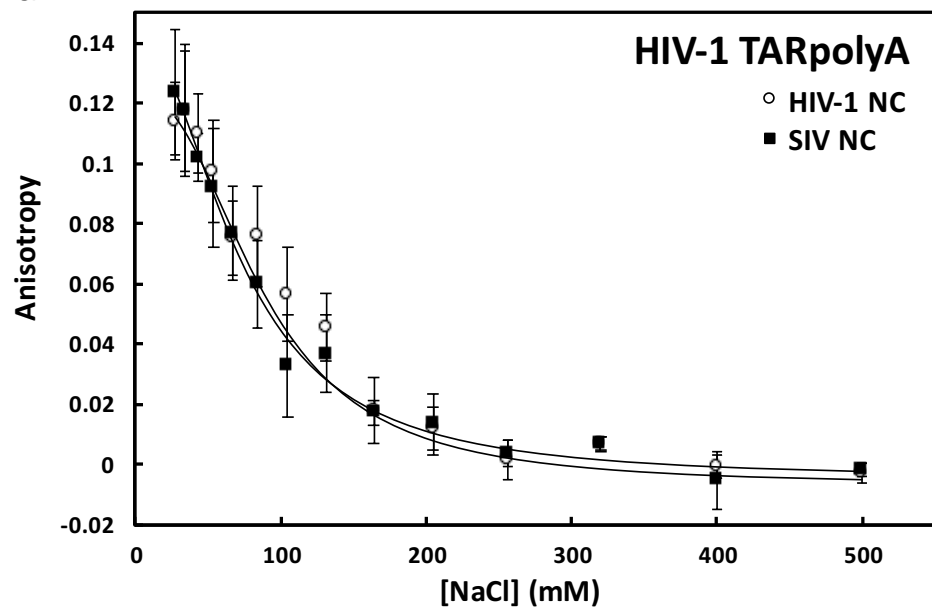

**b**

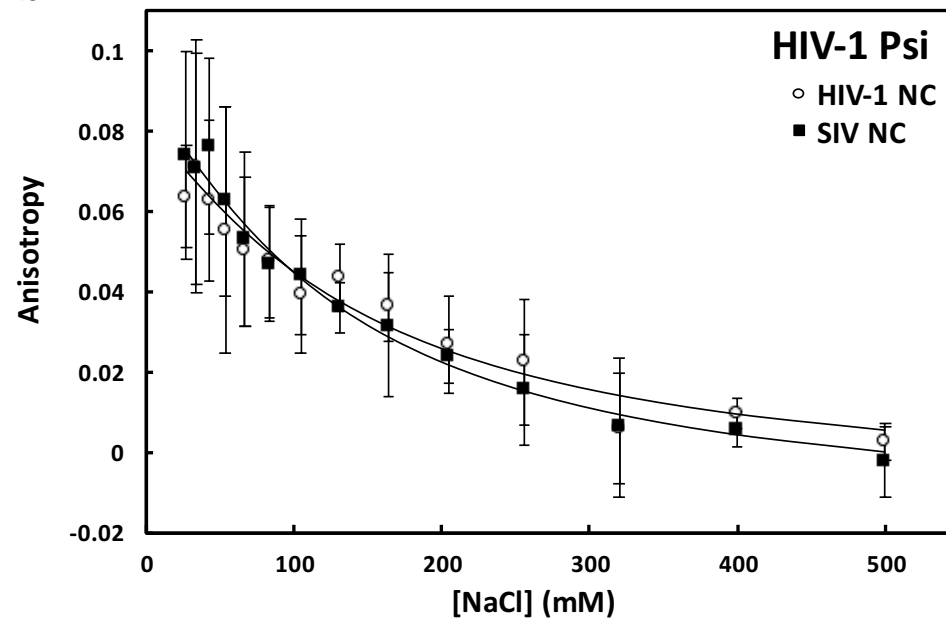

**c**

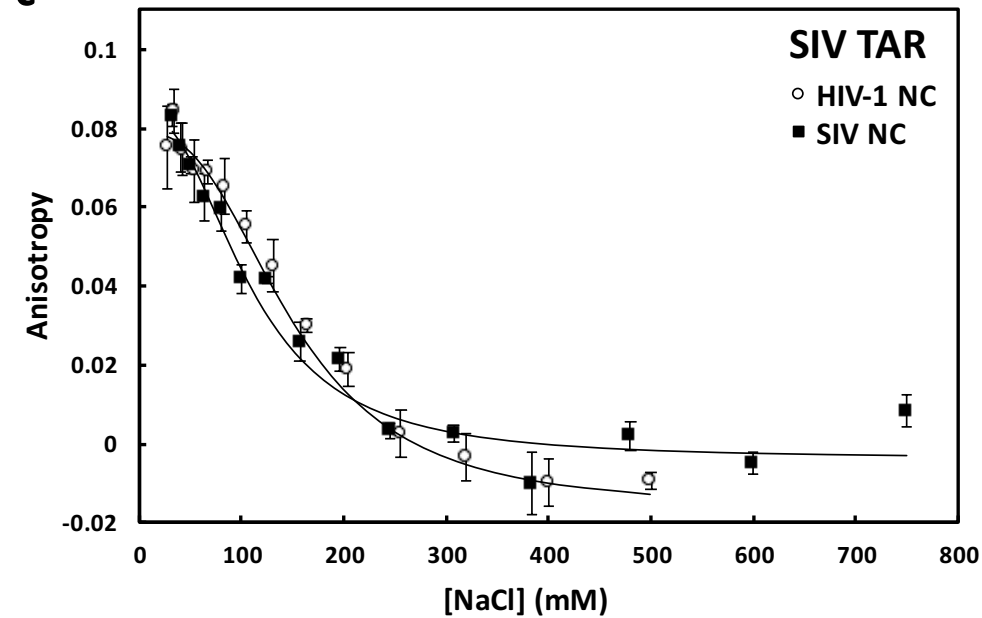

**d**

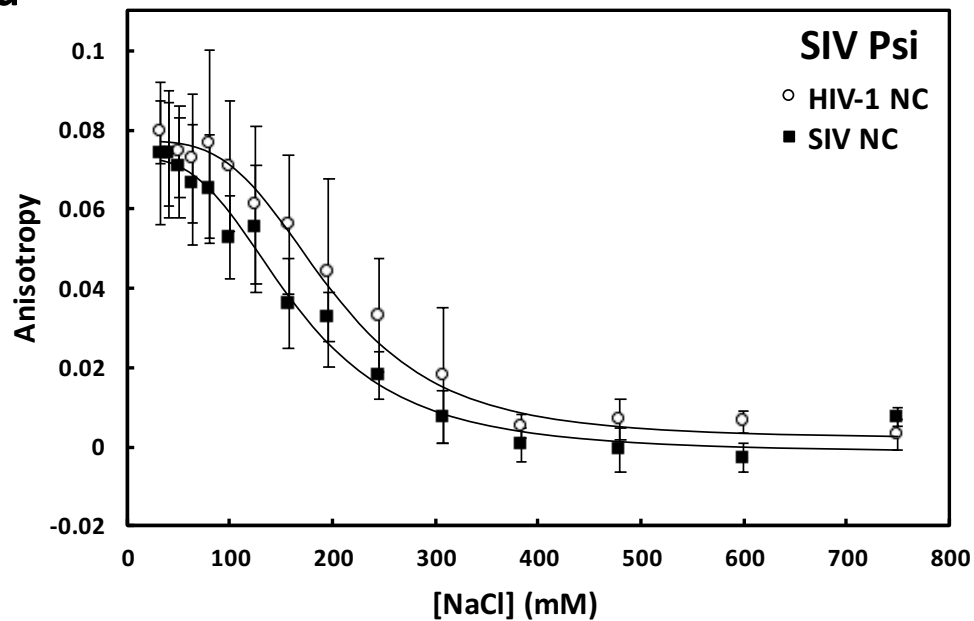

Supplement: Supplementary file 5 — Additional file 5: Fig. S4. Salt-titration binding curves for the interaction of HIV-1 NC and SIV NC with HIV-1 and SIV RNAs. a HIV-1 TARpolyA. b HIV-1 Psi. c SIV TAR. d SIV Psi. Each curve represents the average of at least three independent experiments and the error bars are the standard deviation of the mean. [file 12977_2016_322_MOESM5_ESM.pdf]

Figure S5

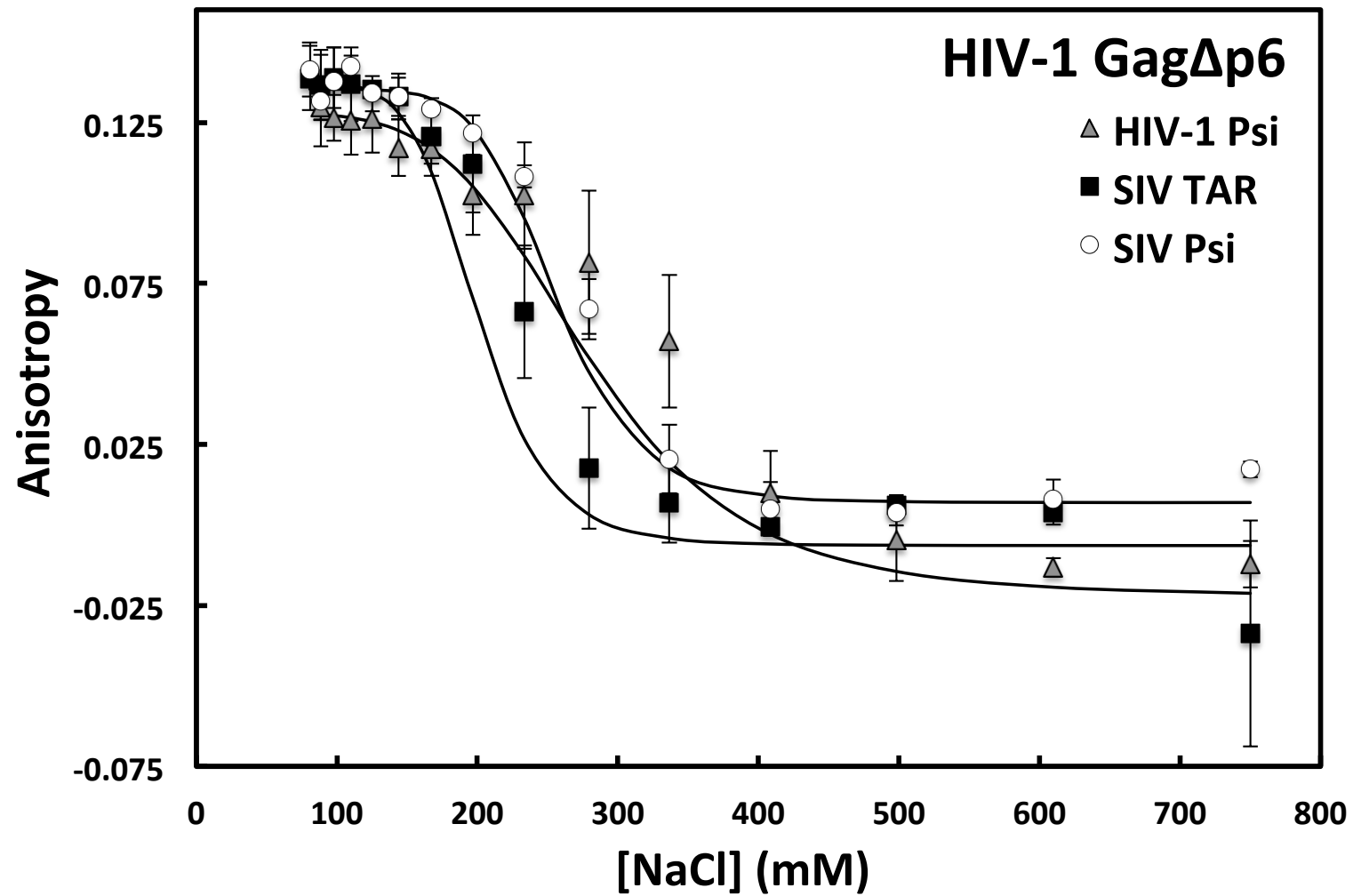

Supplement: Supplementary file 6 — Additional file 6: Fig. S5. Salt-titration binding curves for the interaction of HIV-1 Gag∆p6 with HIV-1 Psi, SIV TAR, and SIV Psi. Each curve represents the average of at least three independent experiments and the error bars are the standard deviation of the mean. [file 12977_2016_322_MOESM6_ESM.pdf]

# Figure S6

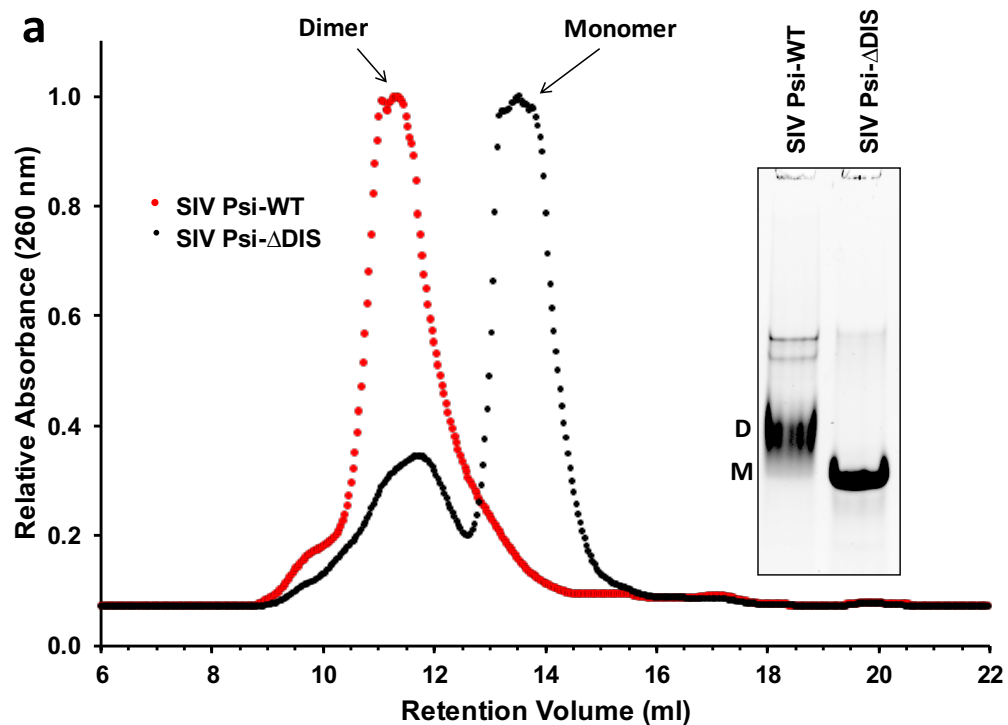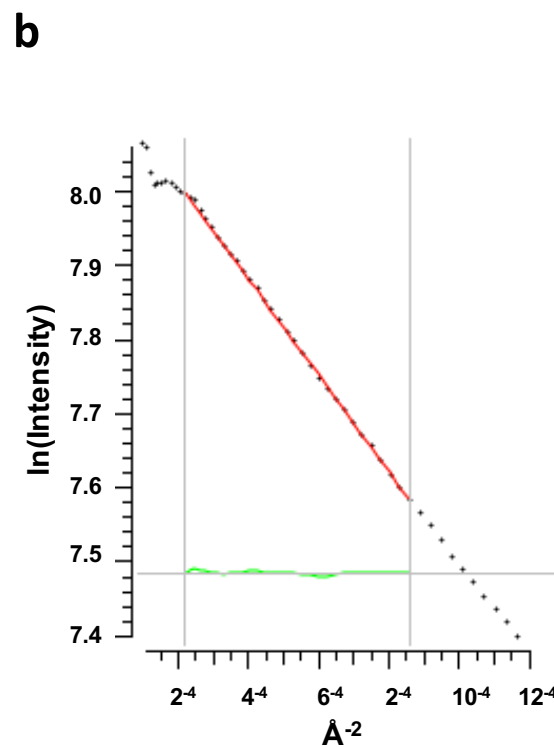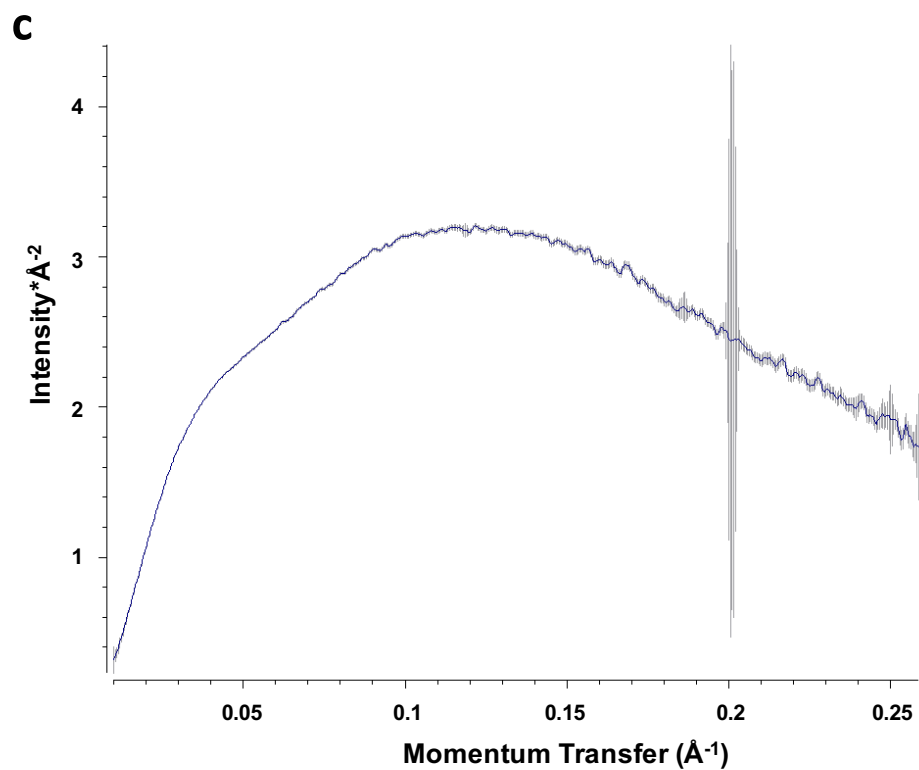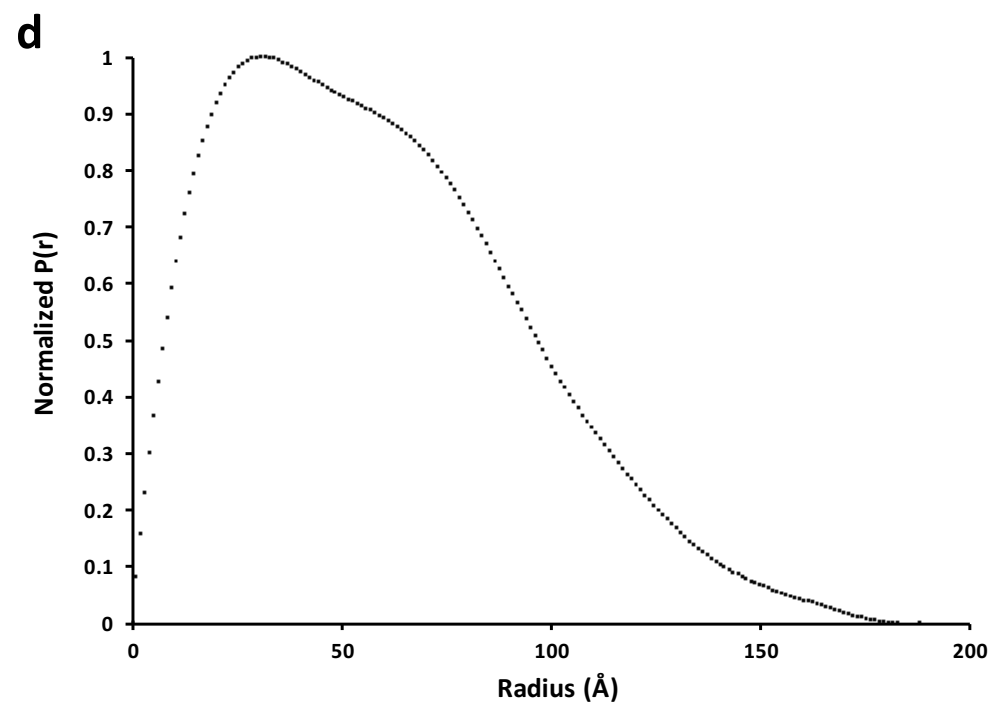

Supplement: Supplementary file 7 — Additional file 7: Fig. S6. Purification and SAXS curve analysis for SIV Psi-∆DIS RNA. a SEC trace of WT (red) and ∆DIS (black) SIV Psi RNAs reveals that mutation of the DIS loop is required to yield the monomeric species. Monomeric SIV Psi RNA was purified using this SEC protocol immediately prior to SAXS data collection. The inset shows analysis of the SEC-purified SIV Psi-∆DIS RNA by native polyacrylamide gel electrophoresis run concurrently with SAXS data collection of the same sample, confirming that the sample used in the SAXS experiment was a homogenous monomer. SIV Psi-WT was run as a control on the same gel, showing that it is predominately a dimer. b The Guinier plot of the low scattering angle (Å−2) region of the SAXS data fits well to a linear regression (red line) with low residuals (green line), indicating that the SIV Psi-∆DIS sample is not aggregated. c The Kratky plot shows a downward trend as momentum transfer/scattering angle increases, indicating that the RNA is nonglobular but well folded. The blue line represents the Kratky transformation of the scattering intensity and the gray bars represent the corresponding error in the intensity measurement. d The P(r) distribution plot indicates that the majority of electron pair distances lie between 25 and 65 Å for this RNA, and that the largest electron pair distance (i.e. Dmax) lies at 188 Å. [file 12977_2016_322_MOESM7_ESM.pdf]

# Figure S7

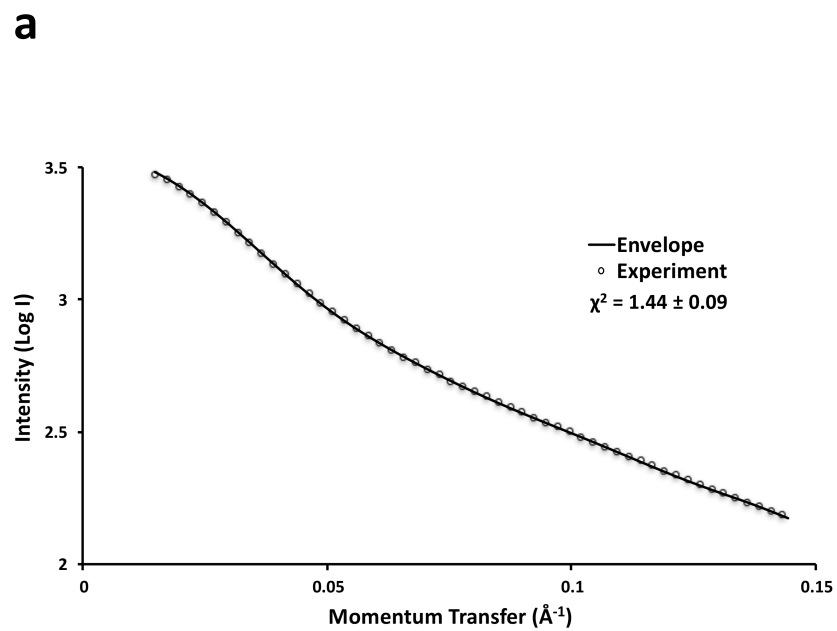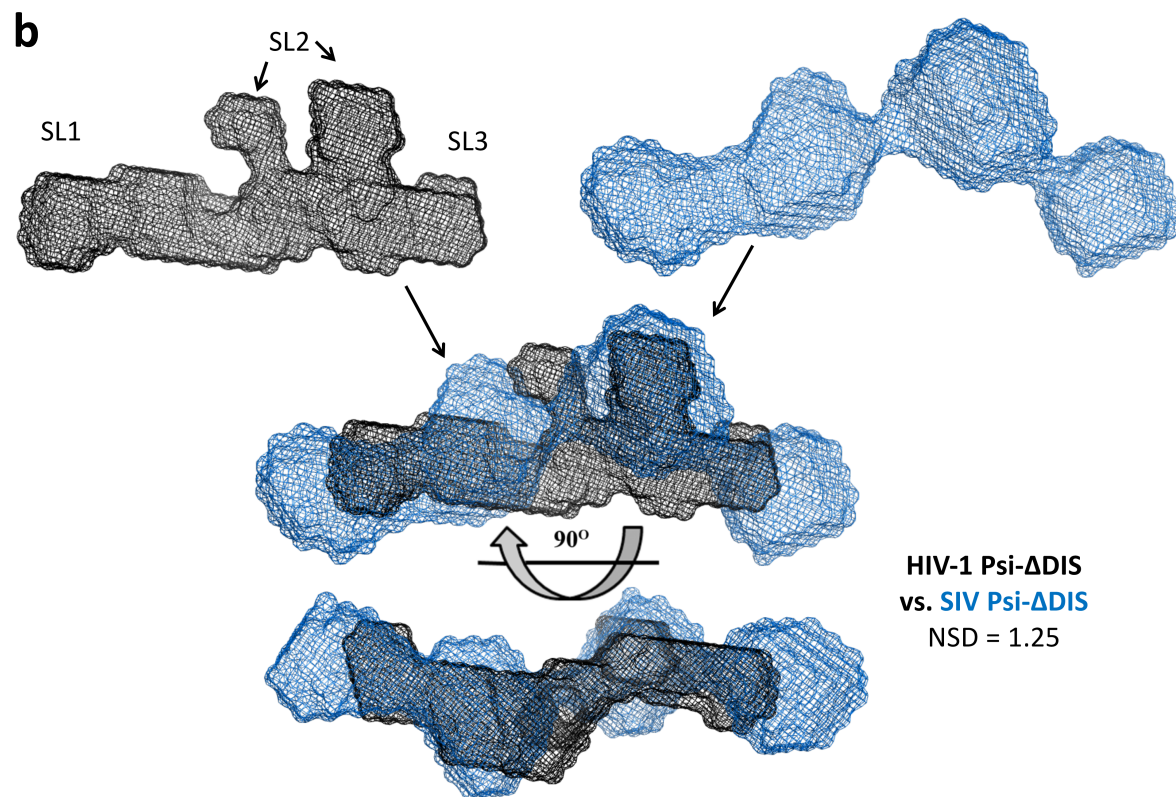

Supplement: Supplementary file 8 — Additional file 8: Fig. S7. SAXS data obtained for SIV Psi-∆DIS RNA (146 nt) and comparison to HIV-1 Psi-∆DIS (105 nt). a Plot of intensity versus momentum transfer for SIV Psi-∆DIS RNA. The open circles indicate every fifth data point from the experimental SAXS curve and the black line represents the back-calculated scattering curve of the ab initio envelope. The χ2 fit between the experimental and back-calculated ab initio envelope scattering curves is reported. b Comparison of the SIV Psi-∆DIS and HIV-1 Psi-∆DIS envelopes showing the confirmed SL structures of the RNAs [28]. The two envelopes were superimposed using the SUPCOMB program [113] and the NSD value for the comparison is indicated. [file 12977_2016_322_MOESM8_ESM.pdf]
