# Supplementary material for: Mechanistic differences between HIV-1 and SIV nucleocapsid proteins and cross-species HIV-1 genomic RNA recognition
Source: Retrovirology. 2016 Dec 29;13:89. doi: 10.1186/s12977-016-0322-5 (PMC5198506; doi:10.1186/s12977-016-0322-5)
Supplement: Supplementary file 4 — Additional file 4. Single molecule methods. [file 12977_2016_322_MOESM4_ESM.pdf]

Additional file 4: Single molecule methods.

The extensible worm-like chain (WLC) describes the theoretical extension $(b_{ds}(F))$ of a dsDNA molecule as a function of force (Additional file 3: Fig. S3, solid gold), where [109],

$$b_{ds}(F) = B_{ds}\left\{1-\frac{1}{2}\left(\frac{k_B T}{FP_{ds}}\right)^{1/2}+\frac{F}{S_{ds}}\right\} \tag{1}$$

The extensible freely-jointed chain (FJC), models the extension of an ssDNA molecule ($b_{ss}(F)$) as a function force F (Additional file 3: Fig. S3, solid pink) [109]:

$$b_{ss}(F) = B_{ss}\left[\coth\left(\frac{2P_{ss}F}{K_B T}\right)-\frac{1}{2}\frac{k_B T}{P_{ss}F}\right]\left[1+\frac{F}{S_{ss}}\right] \tag{2}$$

The typical parameters from our fits for stretch moduli (S_{ds}= 1361 pN, S_{ss}= 720 pN), persistence lengths (P_{ds}= 45 nm, P_{ss}= 0.75 nm), and contour lengths (B_{ds}= 0.34 nm/bp, B_{ss}= 0.55 nm/bp) were used.

To determine the hysteresis area ratio, we find the linear combination ($b_{lin}(F)$) of the WLC and FJC (Additional file 3: Fig. S3, solid red) that intersects the highest data point (b_{max}, F_{max}), where b_{max} is the highest extension reached and F_{max} is the highest force reached:

$$b_{lin}(F) = (1-f)b_{ds}(F) + fb_{ss}(F), \tag{3}$$

where,

$$f = \frac{b_{max} - b_{ds}(F_{max})}{b_{ss}(F_{max}) - b_{ds}(F_{max})} \tag{4}$$

The absolute hysteresis area (H_{abs}) is given by the area between the stretch (Additional file 3: Fig. S3, solid light blue) and return (Additional file 3: Fig. S3, dashed light blue) curves of the force-extension data in the presence of protein (Additional file 3: Fig. S3, filled yellow). The area between the stretch and WLC-FJC linear combination (Additional file 3: Fig. S3, solid red) represents the maximum possible hysteresis area (H_{max}) for a given force-extension curve in the presence of protein (Additional file 3: Fig. S3, filled yellow + filled green). We define the hysteresis area ratio to be,

$$\text{hysteresis ratio} = \frac{H_{abs}}{H_{max}}, \tag{5}$$

which is independent of how far the DNA is stretched for a particular curve. The transition slope (θ) is defined to be the slope of the line describing the force-extension data between the extensions 0.4 nm/bp and 0.5 nm/bp on the dsDNA stretch, in the presence of protein (Additional file 3: Fig. S3, dashed dark blue). The best line is found by fitting the corresponding data points using linear least squares method. We describe the concentration dependence of the transition slope by fitting the data to a simple binding isotherm:

$$\theta(C) = \theta_0 + \frac{(\theta_{max} - \theta_0)}{1 + \frac{K_d}{C}} \tag{6}$$

where C is the protein concentration, θ_0 is the measured transition slope in the absence of protein, and θ_{max} and K_d are fitting parameters that represent the maximum transition slope and the equilibrium dissociation constant, respectively.
