# Supplementary material for: Mechanistic differences between HIV-1 and SIV nucleocapsid proteins and cross-species HIV-1 genomic RNA recognition
Source: Retrovirology. 2016 Dec 29;13:89. doi: 10.1186/s12977-016-0322-5 (PMC5198506; doi:10.1186/s12977-016-0322-5)
Supplement: Supplementary file 9 — Additional file 9: Table S1. Statistics determined from analysis of the SAXS scattering curves and construction of ab initio envelopes using DAMMIN. [file 12977_2016_322_MOESM9_ESM.pdf]

Additional file 9: Table S1. Statistics determined from analysis of the SAXS scattering curves and construction of ab initio envelopes using DAMMIN

**SAXS Parameter**	**SIV Psi-ΔDIS RNA**	**HIV-1 Psi-ΔDIS RNA**
**Scattering Curve**		
[a]**R_g (Å), Guinier**	44 ± 1	[f]33.8 ± 0.4
[a]**R_g (Å), P(r)**	48.5 ± 0.2	[f]34.5
[b]**D_{max} (Å)**	188	[f]121
[c]**DAMMIN, 1st round (*n = 20*)**		
[d]**χ^2**	1.5 ± 0.3	[f]1.14 ± 0.02
[e]**NSD**	0.70 ± 0.05	[f]0.86 ± 0.05
[c]**DAMMIN, 2nd round (*n = 24*)**		
[d]**χ^2**	1.44 ± 0.09	1.36 ± 0.04
[e]**NSD**	0.36 ± 0.03	0.40 ± 0.02

[a]The radius of gyration (R_g) represents the mass distribution about a particle's center of gravity, as calculated from the Guinier plot or P(r) function.
[b]Maximum inter-electron distance (D_{max}) in the RNA as calculated from the P(r) function.
[c]DAMMIN statistics are calculated as the average of the number of iterations run (*n*), and all errors are reported as the standard deviation of this average.
[d]χ^2 reflects the goodness of fit comparison between the experimental scattering curve and the scattering curves back-calculated from the DAMMIN-generated envelopes.
[e]Normalized spatial discrepancy (NSD) is a measure of the overall variance between 3D shapes (in this case ab initio models).
[f]Data taken from Jones et al. [28].
