# Supplementary material for: Mechanistic differences between HIV-1 and SIV nucleocapsid proteins and cross-species HIV-1 genomic RNA recognition
Source: Retrovirology. 2016 Dec 29;13:89. doi: 10.1186/s12977-016-0322-5 (PMC5198506; doi:10.1186/s12977-016-0322-5)
Supplement: Supplementary file 10 — Additional file 10. SAXS data analysis and results. [file 12977_2016_322_MOESM10_ESM.pdf]

Additional file 10: SAXS data analysis and results.

Visual inspection of the SAXS data (Additional file 8: Fig. S7a) and linearity of the Guinier region (Additional file 7: Fig. S6b) indicated that the sample was neither aggregated nor experiencing interparticle repulsions. Furthermore, Kratky analysis of the SIV Psi-ΔDIS suggested that the RNA was well folded and elongated in solution (Additional file 7: Fig. S6c). The radius of gyration (R_g), which represents the mass distribution about a particle's center of gravity, was separately calculated from the slope of the Guinier plot (44 Å) and the pair distance distribution (P(r)) function (48.5 Å), a histogram of all inter-electron distances in the RNA (Additional file 7: Fig. S6d and Additional file 9: Table S1). The maximum electron pair distance (D_{max}) was calculated to be 188 Å from the P(r) function. For reference, the R_g and D_{max} for HIV-1 Psi-ΔDIS were determined to be ~35 Å and 121 Å, respectively, consistent with HIV-1 Psi being shorter and more compact overall than its SIV counterpart [28].

Ab initio envelopes for SIV Psi-ΔDIS were generated as described in the Methods section. After the first round of model generation, the average χ^2 fit to the scattering data was 1.5 and the normalized spatial discrepancy (NSD) between the 20 models was 0.7. NSD is a measure of the overall variance between 3D shapes (in our case the ab initio envelopes), and values <1 are not generally considered to be significantly different. The χ^2 fit and NSD values were improved after the second round of high-density bead ab initio modeling to 1.44 and 0.36, respectively (Additional file 8: Fig. S7a and Additional file 9: Table S1). A second round of high-density bead ab initio modeling was also performed using HIV Psi-ΔDIS SAXS data previously acquired [28], resulting in a χ^2 fit and NSD of 1.36 and 0.40, respectively. This additional refinement step allowed for a more valid comparison between SIV and HIV-1 Psi-ΔDIS structures. The final, averaged ab initio envelopes for HIV-1 and SIV Psi-ΔDIS are shown separately and also overlaid (Additional file 8: Fig. S7b). The NSD value comparing the HIV-1 and SIV envelopes was calculated to be 1.25.

The HIV-1 envelope is similar to the one previously determined (using a single round of model generation), except in the SL2 region where we now observe two regions of density in the final envelope rather than one. The two features obtained for SL2 may be due to structural heterogeneity in this region, which was previously shown to be the least well-defined SL based on comparison of an all-atom model to a SAXS envelope [28]. The second round of refinement

carried out here reveals two distinct protrusions, which may represent alternative structures that can form.
